# Interfacial Polysulfide Confinement via Spatially Controlled Sulfonated Metal–Organic Polyhedra Coatings in Lithium–Sulfur Batteries

**DOI:** 10.1002/advs.75347

**Published:** 2026-04-16

**Authors:** Soyeon Ko, WooYeon Moon, Yuwei Zhu, Yuhui An, Yunyuan Lu, Sourayan Bal, Linqin Mu, Kyung Min Choi, Yoon Hwa

**Affiliations:** ^1^ Chemical Engineering Fulton Schools of Engineering Arizona State University Tempe Arizona USA; ^2^ Department of Chemical and Biological Engineering Sookmyung Women's University Seoul Republic of Korea; ^3^ Materials Science and Engineering Fulton Schools of Engineering Arizona State University Tempe Arizona USA; ^4^ School of Electrical Computer and Energy Engineering Arizona State University Tempe Arizona USA

**Keywords:** functional coating, lithium polysulfide, lithium–sulfur batteries, metal–organic polyhedra, sulfur‐carbon composite

## Abstract

Lithium‐sulfur (Li‐S) batteries offer a high theoretical specific energy of 2600 Wh kg^−1^, yet commercialization remains limited by poor sulfur conductivity and structural instability of sulfur electrodes due to polysulfide (Li‐PS) dissolution and shuttling. Here, a sulfonated zirconium‐based metal organic polyhedron (SMOP) is introduced as a molecularly dispersible additive for nanoscale interfacial engineering at 3 wt.% in the sulfur–carbon composite. Two placement modes are implemented, where SMOP is assembled as a conformal interfacial coating on sulfur‐loaded hollow carbon spheres (SMOP‐S‐HCS), whereas SMOP is introduced by co‐deposition/physical blending with Ketjen black (SMOP‐S‐KB), producing a dispersed/buried distribution without a defined surface layer. SMOP‐S‐HCS delivers a low shuttle charge (*Q_PS_*) of 54.0 mAh g_S_^−1^, whereas SMOP‐S‐KB exhibits approximately 1.7‐fold higher *Q_PS_* despite identical SMOP loading during the Li‐PS shuttle current measurement. SMOP‐S‐HCS delivers robust rate capability with discharge capacity ranging from ∼1500 to ∼800 mAh g_S_^−1^ from 0.05 to 2C and sustained discharge capacity of 850 mAh g_S_^−1^ after 400 cycles at 0.3C. Ex situ sulfur K‐edge XAS confirms the effectiveness of SMOP functional barrier against Li‐PS shuttling by probing more reversible sulfur speciation and reduced oxidized sulfur buildup during cycling.

## Graphical abstract

Spatially controlled sulfonated metal–organic polyhedra (SMOP) coating on sulfur‐loaded hollow carbon spheres form ultrathin, conformal interfacial barriers that selectively confine soluble polysulfides, stabilize sulfur redox, and suppress shuttle reactions. In contrast, the same SMOP content dispersed within sulfur–Ketjen black aggregates remains buried, unable to effectively mitigate polysulfide shuttling, resulting in persistent shuttle‐induced capacity loss and reduced electrochemical stability.

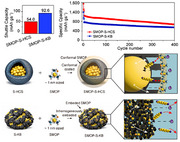

## 1 Introduction

A lithium‐sulfur (Li‐S) battery is widely regarded as one of the most promising next‐generation rechargeable batteries due to its high theoretical specific energy (2600 Wh kg^−1^) which significantly exceeds that of conventional lithium‐ion batteries (∼550 Wh kg^−1^) [1, 2]. In addition, sulfur is earth‐abundant and inexpensive, making Li‐S chemistry attractive for large‐scale energy storage applications [3, 4]. However, the practical cell level energy of Li‐S batteries under realistic operating conditions remain limited mainly due to low electrical conductivity of sulfur [5, 6], the large volumetric change (≈80% for S ↔ Li_2_S) of sulfur [7, 8], and most critically, the formation and uncontrolled migration of lithium polysulfide (Li‐PS) intermediate in organic electrolytes [9]. This phenomenon, so‐called Li‐PS shuttle, leads to loss of active material from the sulfur positive electrode, and the formation of an electrically insulating Li_2_S layer on the lithium negative electrode, resulting in low coulombic efficiency and rapid capacity fading during charge‐discharge cycling. To mitigate Li‐PS shuttling and improve the electrochemical and mechanical stability of Li‐S cells, a variety of approaches have been explored, such as confining sulfur within porous carbon [10, 11], incorporating functional metal oxides or sulfides [12, 13], employing functional polymer binder [14, 15], and employing functionalized separators [16, 17, 18]. Porous carbons are hardly dispensable for establishing electronic percolation and accommodating sulfur species, but carbon‐based architectures alone generally provide limited chemical affinity toward Li‐PS species and thus cannot fully prevent their dissolution and migration. This has motivated the incorporation of chemically functional components that can more strongly interact with soluble polysulfides while minimizing the penalty of inactive mass [19, 20, 21].

In this regard, metal‐organic polyhedra (MOPs), ∼1.0 nm‐sized discrete cage‐like coordination assemblies constructed from metal nodes and organic linkers, offer a distinct opportunity to overcome aforementioned limitations [22, 23]. Their solution processability, structural precision, and modular synthesis allow dense incorporation of targeted functional groups (e.g., sulfonate, phosphonate, amine) directly onto the cage backbone [24, 25]. Although conceptually related to metal‐organic frameworks (MOFs) [26, 27, 28, 29] or covalent organic frameworks (COF) [18, 30]. MOPs exist as molecular entities rather than extended lattices, allowing molecular‐level dispersion to form ultrathin (∼1 nm) coatings or interfacial layers, dramatically increasing the effective functional surface area per its unit mass [31]. This feature is particularly attractive for Li‐S electrodes because it creates the possibility of achieving meaningful polysulfide regulation with only a very small amount of functional material. In contrast to previously reported MOF‐related sulfur‐electrode strategies, which often require substantially larger fractions of framework‐related material (>10 wt.%) on the full‐electrode basis [32, 33, 34], MOPs can, in principle, provide a high density of chemically active interfacial sites at much lower loading, thereby minimizing penalties in electrode energy density and cost [31]. In sulfur‐carbon composites, such thin interfacial layers are expected to work synergistically with the conductive carbon framework by introducing chemically selective sites [35, 36], while preserving electronic percolation. Under this constraint, not only the chemistry of the MOP but also how such a small amount of MOP is spatially deployed is expected to be critical for maximizing its effectiveness.

In this work, we introduce a sulfonated zirconium (Zr)‐based metal‐organic polyhedron (SMOP, [Cp_3_Zr_3_O(OH)_3_]_4_[BDC]_6_[(C_2_H_5_)_2_NH_2_]_2_Cl_6_]) as a functional additive for sulfur positive electrodes. SMOP integrates the robust Zr‐O framework with sulfonate (‐SO_3_^−^) groups, offering chemical stability [37] and ionically functional surface that can interact Li‐PS in organic liquid electrolytes. In particular, sulfonate moieties can coordinate Li^+^ in Li‐PS species, thereby forming a chemically selective interfacial barrier that suppresses Li‐PS shuttling [38, 39, 40]. Because these functional groups are presented on the surface of each discrete cage, SMOP is expected to deliver substantial interfacial functionality even at very low loading. A key question, however, is how to deploy such a small amount of SMOP so that its chemical affinity is translated into the greatest cathode‐level benefit. Simply dispersing SMOP throughout the electrode may underutilize its interfacial role, whereas controlled placement at sulfur‐carbon surfaces may enable a much higher functional efficiency per unit mass of additive. Motivated by this idea, we examine spatial placement as a central design parameter by comparing two representative carbon hosts with contrasting sulfur accommodation and transport characteristics and by implementing two deployment modes: conformal SMOP coating on sulfur‐carbon composites and physical blending of SMOP into the electrode. This head‐to‐head design allows us to determine how a minimally added, molecularly dispersible MOP can be most effectively positioned to regulate polysulfides and improve sulfur‐electrode performance.

## 2 Results and Discussion

**FIGURE 1:**
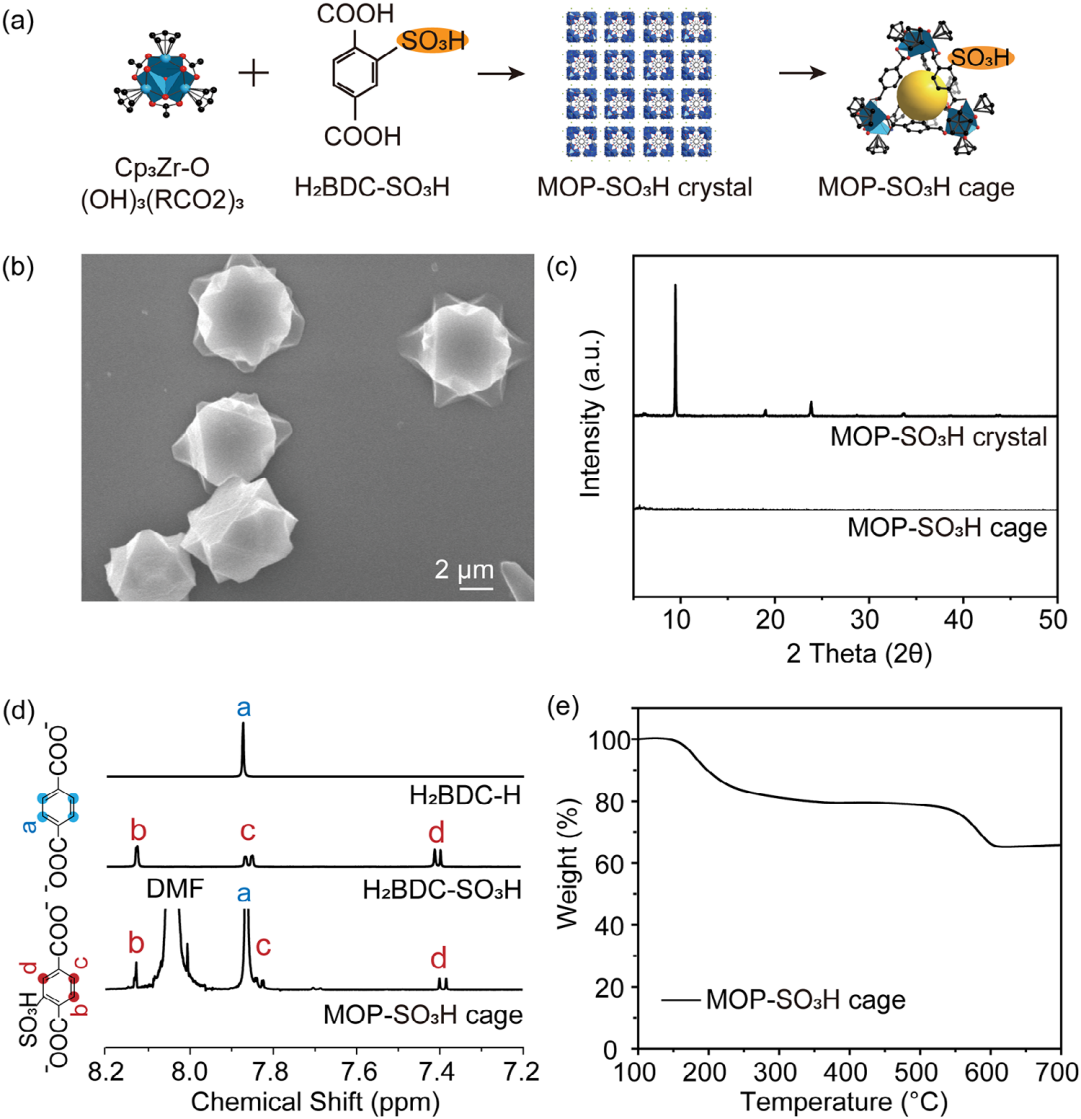
Structural and compositional characterization of synthesized SMOP. (a) Schematic illustration of the Zr‐based SMOP structure and crystal‐to‐cage transformation. (b) SEM images of a well‐defined cubic SMOP crystal. (c) XRD patterns of SMOP crystal and molecularly dispersed SMOP cages. (d) ^1^H NMR spectra of terephthalic acid, sulfonated terephthalic acid, and the SMOP cage. Peak a (7.85 ppm) is assigned to unsubstituted terephthalate (H_2_BDC‐H), whereas peaks b (8.07 ppm), c (7.84 ppm), and d (7.41 ppm) correspond to sulfonated terephthalate (H_2_BDC‐SO_3_H). (e) TGA curve of SMOP cage under He atmosphere.

To enable the two deployment modes of SMOP in sulfur‐carbon composites, such as solution‐mediated conformal coating and physical blending, we first synthesized a Zr‐based SMOP and established its crystal‐to‐cage transition, which underpins its molecular dispersibility and solution processability. As illustrated in Figure 1a, SMOP was prepared by solvothermal reaction of Cp_2_ZrCl_2_ and 2‐hydroxyterephthalic acid (H_2_BDC‐SO_3_H), yielding a crystalline product composed of periodically arranged SMOP cages. Based on the established geometry of analogous tetrahedral Zr‐based MOPs, the characteristic molecular dimension of an individual SMOP cage is expected to be on the order of ∼1–1.5 nm [41]. The scanning electron microscopy (SEM) image of the as‐synthesized SMOP crystal exhibits highly crystalline particles with uniform cubic morphology, confirming the successful formation of an extended lattice of discrete SMOP nanocages. Upon immersion in methanol (MeOH), these crystals readily dissociate into discrete SMOP cages because hydrogen bonds between adjacent nanocages are replaced by stronger MeOH‐cage interactions, giving molecularly dispersed SMOP cages [35, 36]. This crystal‐to‐cage transition is corroborated by X‐ray diffraction (XRD) (Figure 1c), where sharp diffraction peaks of SMOP crystal demonstrate its ordered framework structure, whereas the absence of distinct diffraction peaks in the discrete SMOP cages sample indicates its non‐crystalline, molecularly dispersed state [41]. This behavior is critical for interfacial engineering, as it enables SMOP to be delivered from solution and assembled into ultrathin, coherent interfacial layers on suitable sulfur–carbon surfaces.

The incorporation of both unsubstituted and sulfonated terephthalate ligands in SMOP was verified by proton nuclear magnetic resonance (^1^H NMR) spectroscopy (Figure 1d). The SMOP crystals were digested in deuterated dimethyl sulfoxide (DMSO‐d_6_) with a strong acid to cleave coordination bonds, yielding well‐resolved aromatic signals. A resonance at 7.85 ppm is assigned to unsubstituted terephthalate (H_2_BDC‐H), while signals at 8.07, 7.84, and 7.41 ppm are assigned to sulfonated terephthalate (H_2_BDC‐SO_3_H) [37]. The signal observed at 8.02 ppm is attributed to residual DMF from the synthesis. The simultaneous presence of both sets of peaks confirms incorporation of a H_2_BDC‐H to H_2_BDC‐SO_3_H linkers, and integration of these signals indicates a ratio of approximately 5:1 per SMOP cage between H_2_BDC‐H and H_2_BDC‐SO_3_H. The thermal characteristics of SMOP were examined by thermogravimetric analysis (TGA) under helium (He) at a heating rate of 20°C min^−1^ (Figure 1e). A weight loss near 200°C corresponds to the decomposition of sulfonic acid groups in the SMOP ligands [42], followed by a subsequent weight loss above 500°C associated with the decomposition of the organic linkers, similar to the behavior of UiO‐66 [43]. Above 600°C, the mass remains nearly constant, indicating the formation of a thermally stable Zr‐O residue [44]. This behavior confirms that SMOP is thermally stable far beyond the typical operating temperature window of Li‐S batteries (60°C).

To investigate how incorporation and spatial placement of SMOP within the sulfur‐carbon composite governs the electrochemical behavior of sulfur positive electrodes, sulfur‐carbon composites were prepared using two structurally distinct carbon hosts, such as hollow carbon spheres (HCS, SEM image is shown in Figure S1) and ketjen black (KB, SEM image shown in Figure S2), and different SMOP deposition strategies in sulfur‐carbon composites. HCS particles (∼200 nm) comprise a central macropore cavity surrounded by a mesoporous shell, which allows sulfur to be incorporated into the hollow interior and, once loaded, substantially shields the incorporated sulfur from direct exposure to the external environment [45, 46]. Sulfur‐loaded HCS (S‐HCS) was synthesized by a chemical precipitation method, in which sulfur was dissolved in carbon disulfide (CS_2_), and isopropanol (IPA) was added as a co‐solvent to facilitate infiltration of the sulfur solution into the hollow cavity [47]. Subsequent evaporation of CS_2_ and IPA yielded the S‐HCS composite. The resulting S‐HCS was then coated with SMOP (denoted SMOP‐S‐HCS) to form an outer barrier layer intended to immobilize Li‐PS generated from sulfur confined within the HCS cavity during cycling.

**FIGURE 2:**
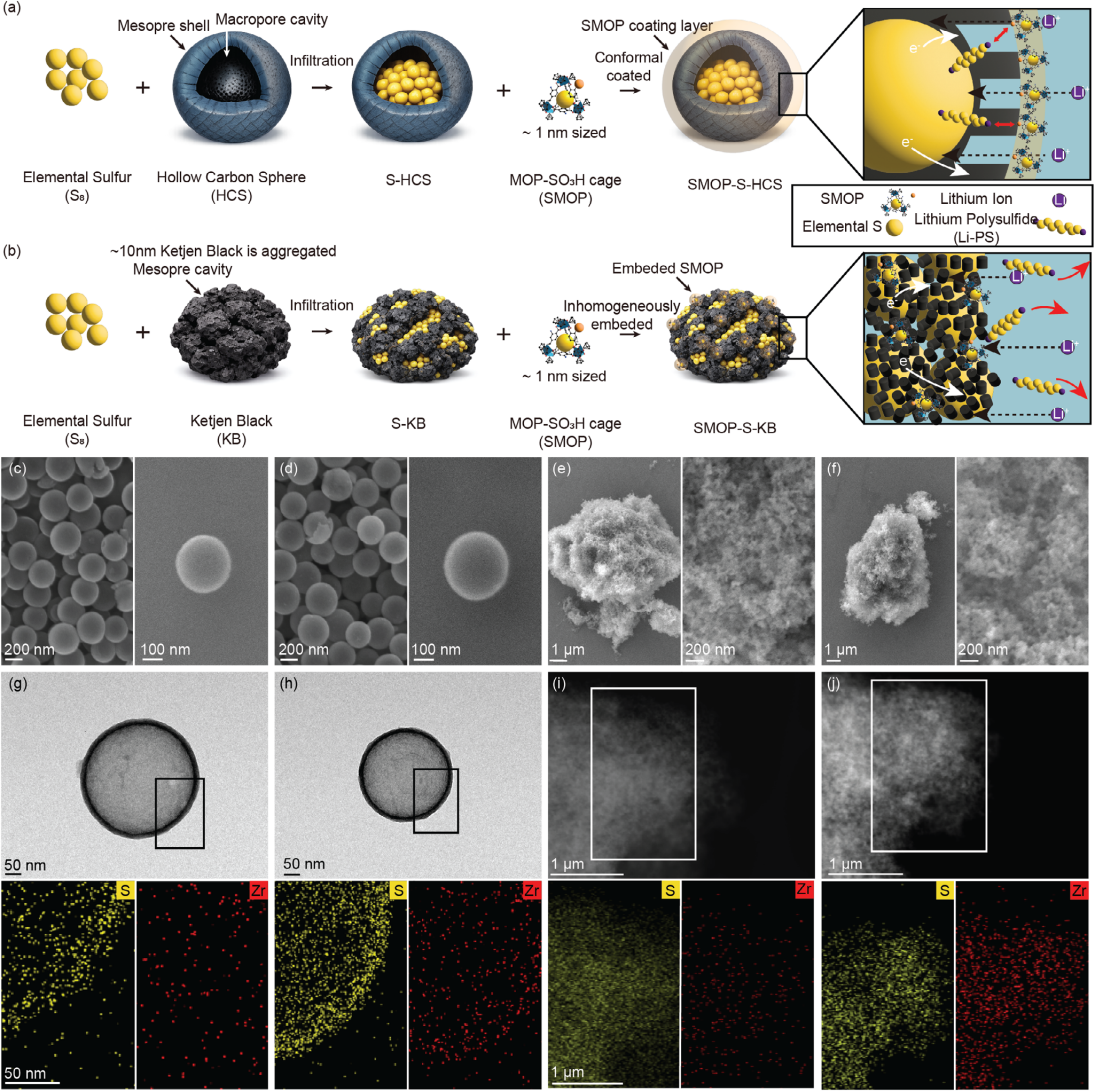
Morphology and elemental distribution of S‐HCS, SMOP‐S‐HCS, S‐KB, and SMOP‐S‐KB. Schematic illustration of sulfur encapsulation and SMOP surface modification for (a) SMOP‐S‐HCS and (b) SMOP‐S‐KB, highlighting Li^+^ diffusion, electron transport pathways, and lithium polysulfide confinement by SMOP cages. SEM images of (c) S‐HCS, (d) SMOP‐S‐HCS, (e) S‐KB, and (f) SMOP‐S‐KB. TEM image with STEM‐EDS elemental maps of sulfur (S) and zirconium (Zr) for (g) S‐HCS, (h) SMOP‐S‐HCS, (i) S‐KB and (j) SMOP‐S‐KB.

In contrast, KB consists of nano‐particles (∼10 nm) with predominantly mesoporous character arising from interparticle voids [48], so sulfur preferentially deposits on or between the nanoparticles and remains relatively exposed to the surroundings, providing more accessible pathways for Li‐PS formation and shuttling. For the KB system, sulfur‐KB composites (S‐KB) were prepared by mixing a sulfur‐dissolved CS_2_ solution with KB, followed by solvent evaporation. When SMOP was added simultaneously with sulfur in CS_2_, the resulting composite (SMOP‐S‐KB) contained SMOP randomly dispersed throughout the composite particles rather than forming a defined coating. According to TGA, the sulfur content in the as‐prepared S‐HCS and S‐KB composites is ∼78 wt.% (Figure S3). SMOP was subsequently introduced by solvent evaporation at a fixed sulfur‐carbon composite: SMOP mass ratio of 97:3 (*w/w*), corresponding to 3 wt.% SMOP in the final composite. Accordingly, the sulfur content in the SMOP‐containing composites is ∼75.7 wt.%, assuming quantitative SMOP retention and unchanged sulfur loading. A schematic illustration of these architectures and the associated Li^+^ transport, electron pathways, and Li‐PS confinement mechanism is provided in Figure 2a,b.

The morphologies of S‐HCS, SMOP‐S‐HCS, S‐KB, and SMOP‐S‐KB are characterized using electron microscopies. In SEM images, S‐HCS (Figure 2c) retains the pristine HCS morphology, including the ∼200 nm particle diameter and smooth, crack‐free surfaces, indicating successful sulfur infiltration into the HCS cavity without structural collapse or the formation of sulfur agglomerates. SMOP‐S‐HCS (Figure 2d) likewise exhibits spherical particles with smooth surfaces and no discernible surface aggregates, consistent with a thin, conformal SMOP coating on the HCS exterior. In contrast, the primary KB nanoparticles in S‐KB (Figure 2e) assemble into micron‐scale secondary aggregates as sulfur fills interparticle voids and bridges adjacent particles. SMOP‐S‐KB (Figure 2f) shows a similar aggregated morphology, and SMOP cannot be directly resolved in SEM due to its molecular‐scale size (∼1.5 nm).

The spatial distributions of sulfur and SMOP were further examined by bright‐field transmission electron microscopy (TEM) and scanning transmission electron microscopy (STEM) coupled with energy‐dispersive X‐ray spectroscopy (EDS) elemental mapping (Figure 2g–j). For S‐HCS (Figure 2g), the bright‐field TEM image reveals a well‐defined carbon shell, and the EDS sulfur map confirms that sulfur is predominantly confined within the HCS interior cavity. For SMOP‐S‐HCS (Figure 2h), sulfur remains localized in the hollow core, whereas the Zr signal is concentrated along the outer shell, directly evidencing a uniform SMOP coating that preserves the encapsulated sulfur. In the KB‐based composites, bright‐field TEM images of S‐KB and SMOP‐S‐KB (Figure 2i,j) show featureless aggregates, and sulfur mapping indicates a homogeneous sulfur distribution throughout the secondary particles. In SMOP‐S‐KB, Zr signals are also dispersed throughout the KB host together with sulfur, consistent with a mixed, but not noncoating architecture. These observations verify that SMOP is incorporated as designed in both architectures, as a surface coating on HCS and as a dispersed additive within KB, thereby achieving the targeted spatial configurations.

**FIGURE 3:**
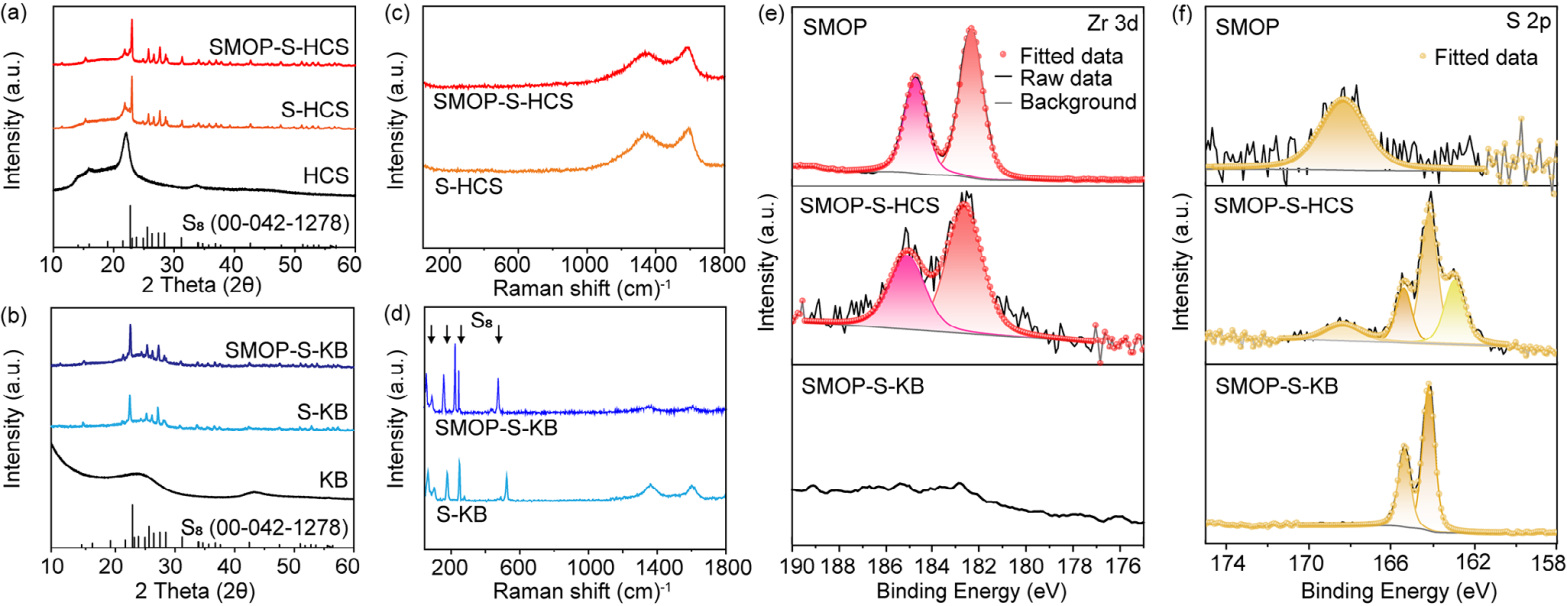
Structural and surface characterization of synthesized S‐HCS, SMOP‐S‐HCS, S‐KB, and SMOP‐S‐KB. (a) XRD patterns of HCS, S‐HCS, and SMOP‐S‐HCS. (b) XRD patterns of KB, S‐KB, and SMOP‐S‐KB. The diffraction pattern of crystalline S_8_ (00‐042‐1278) is shown as a reference. (c) Raman spectra of S‐HCS and SMOP‐S‐HCS. (d) Raman spectra of S‐KB and SMOP‐S‐KB. High‐resolution (e) Zr 3d and (f) S 2p XPS spectra of SMOP, SMOP‐S‐HCS, and SMOP‐S‐KB. All high‐resolution XPS spectra were normalized to their respective maximum intensities to facilitate comparison of peak shapes (not absolute signal intensities).

The structural differences among the composites were first evaluated by X‐ray diffraction (XRD, Figure 3a,b). Pristine HCS and KB exhibit broad diffraction features characteristic of amorphous carbon [49], whereas S‐HCS and S‐KB display additional sharp reflections that match crystalline sulfur (JCPDS 08–0247), confirming successful sulfur incorporation into both carbon hosts. The XRD patterns of SMOP‐S‐HCS and SMOP‐S‐KB are essentially identical to those of S‐HCS and S‐KB, respectively, consistent with the absence of discernible SMOP reflections due to its low loading (3 wt.%) and molecular‐scale, noncrystalline (or highly dispersed) nature. Raman spectra of S‐HCS, SMOP‐S‐HCS, S‐KB, and SMOP‐S‐KB (Figure 3c,d) show broad D and G bands characteristic of disordered sp^2^ carbon in both HCS‐ and KB‐based electrodes. The overall D/G intensity (*I_D_/I_G_*) ratio remains similar before and after SMOP incorporation (S‐KB: 0.85, SMOP‐S‐KB: 0.84, S‐HCS: 0.84, SMOP‐S‐HCS: 0.84), indicating that the crystallographic frameworks of the carbon host are not significantly altered by sulfur loading or SMOP coating. In the KB‐based composites, additional sharp features attributable to crystalline sulfur are observed, whereas the HCS‐based electrodes show only broad backgrounds with no distinct sulfur modes, probably due to the fact that sulfur is confined inside the HCS cavities [50]. These observations support the structural picture inferred from TEM and XRD that sulfur is more surface‐exposed in KB and more effectively encapsulated in HCS.

Consistent with this interpretation, N_2_ physisorption measurements reveal a pronounced loss of gas‐accessible porosity upon sulfur incorporation. Pristine KB and HCS exhibit typical micro/mesoporous adsorption behavior (Figure S4) with high BET surface areas of 1305.23 and 1378.44 m^2^ g^−1^, respectively (Table S1). In contrast, all sulfur‐loaded composites (S‐HCS, SMOP‐S‐HCS, S‐KB, and SMOP‐S‐KB) show a markedly reduced N_2_ uptake over the entire relative‐pressure range (Figure S5), indicating extensive pore filling and/or blocked pore access after sulfur loading. In particular, the low‐pressure uptake (*p/p_o_* < 0.05) and the mesopore‐associated hysteresis are strongly suppressed, evidencing a substantial reduction in accessible micro/mesopore volume. The remaining adsorption near *p/p_o_* approaches to 1 is mainly attributed to interparticle voids. Accordingly, the BET surface areas decreased to 8.66 and 18.93 m^2^ g^−1^ for S‐HCS and S‐KB, respectively (Table S1). Incorporation of SMOP (3 wt.%) results in modest increases to 14.94 m^2^ g^−1^ for SMOP‐S‐HCS and 23.42 m^2^ g^−1^ for SMOP‐S‐KB, while the overall isotherm profiles remain essentially unchanged relative to their SMOP‐free counterparts. This indicates that, at such a low loading, SMOP primarily contributes its intrinsic surface area, accounting for the modest increase in the measured BET surface area. The pore size distribution (PSD) analysis further supports this conclusion. Pristine HCS and KB display distinct mesopore signatures (Figure S6), reflecting their different host architectures. After sulfur incorporation, the PSD results of all sulfur‐loaded composites show strongly suppressed differential pore volume across the measured pore‐width range (Figure S7), and the mesoporous features of HCS‐ and KB‐derived composites become largely indistinguishable. Notably, SMOP‐S‐HCS exhibits a small but discernible PSD feature centered near ∼6 nm, which is consistent with SMOP being preferentially enriched at the outer surface of the S‐HCS particles. In contrast, SMOP‐S‐KB does not show a comparable mesopore feature, consistent with SMOP being largely buried within sulfur‐KB aggregates.

To probe the surface composition and SMOP surface enrichment in the composites, X‐ray photoelectron spectroscopy (XPS) was performed on the SMOP‐containing samples. The survey spectra (Figure S8) are dominated by C 1s and O 1s signals for both composites. Notably, Zr‐related features are clearly observed for SMOP‐S‐HCS, whereas they are barely discernible for SMOP‐S‐KB, indicating substantially greater SMOP exposure at the outermost surface of the HCS‐based composite. This trend is corroborated by the high‐resolution Zr 3d region (Figure 3e), where SMOP‐S‐HCS exhibits a pronounced Zr 3d doublet, while SMOP‐S‐KB shows only a weak/near‐background Zr signal. In the S 2p region (Figure 3f and Figure S8), SMOP‐S‐KB displays an intense, well‐resolved elemental‐sulfur doublet, confirming that sulfur remains highly surface‐accessible in the KB‐based architecture, whereas SMOP‐S‐HCS exhibits a broader S 2p envelope, consistent with sulfur residing beneath and/or interacting with an SMOP‐enriched surface layer. A minor contribution near ∼163 eV in SMOP‐S‐HCS is consistent with reduced sulfur environments (e.g., polysulfide/organic sulfide‐like species) rather than oxidized sulfonate sulfur.

**FIGURE 4:**
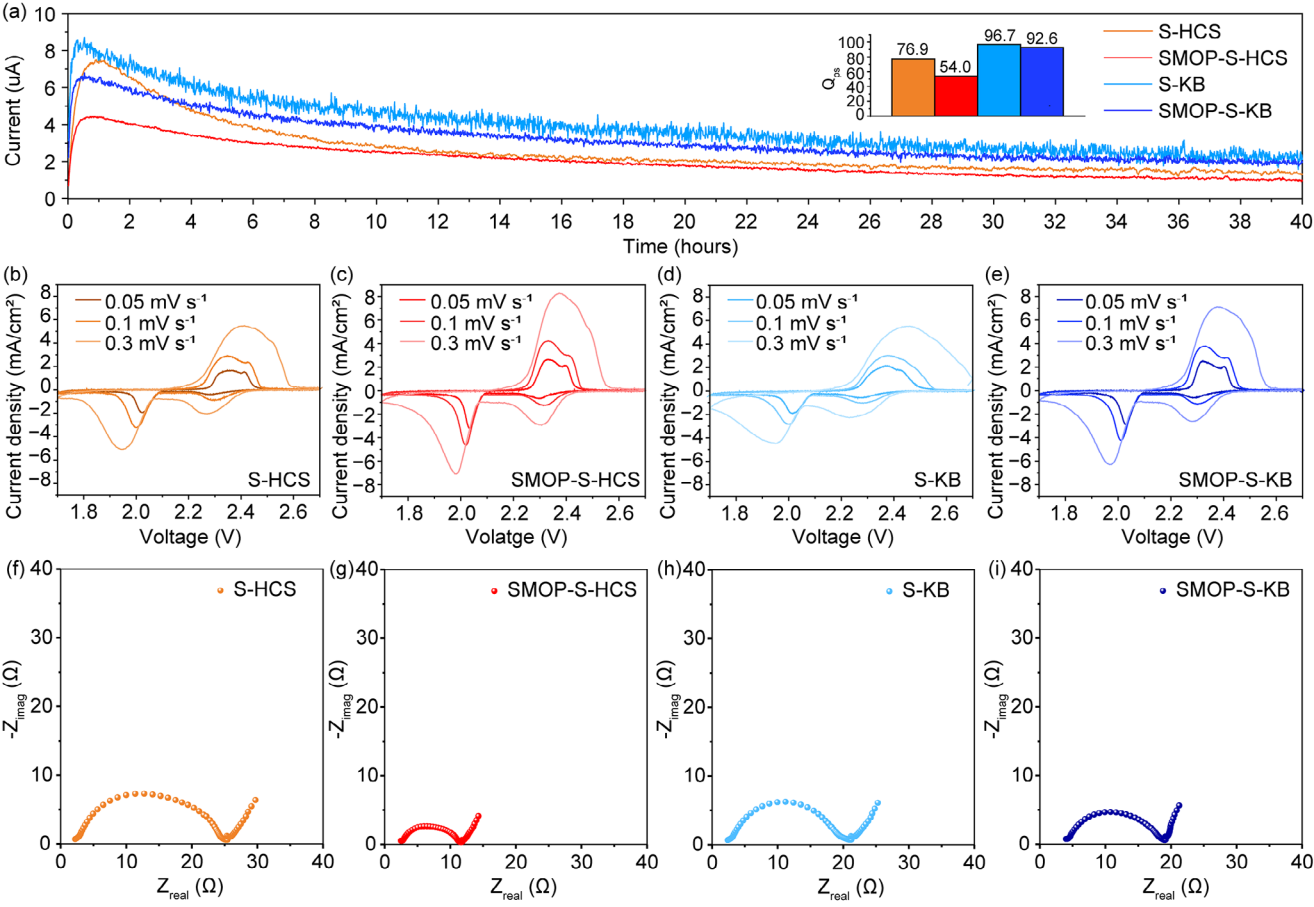
Electrochemical characterization of S‐HCS, SMOP‐S‐HCS, S‐KB, and SMOP‐S‐KB positive electrodes. (a) Chronoamperometrically measured Li‐PS shuttle current collected after the cells were brought to 70% SOC and monitored for 40 h. The corresponding *Q_ps_* values for each electrode are indicated in the inset bar graph. CV curves of (b) S‐HCS, (c) SMOP‐S‐HCS, (d) S‐KB, and (e) SMOP‐S‐KB positive electrodes at various scan rates. Nyquist plots of (f) S‐HCS, (g) SMOP‐S‐HCS, (h) S‐KB, and (i) SMOP‐S‐KB positive electrodes after the CV tests. Areal sulfur loading of the electrodes is 1.5 mg_S_ cm^−2^ and SMOP content in the SMOP‐containing electrodes is 1.8 wt.%.

To elucidate how the incorporation of sulfur and SMOP in the composites affects electrochemical behavior, Li‐S cells with S‐HCS, SMOP‐S‐HCS, S‐KB, and SMOP‐S‐KB positive electrodes were investigated by shuttle‐current measurements, cyclic‐voltammetry (CV), and electrochemical impedance spectroscopy (Figure 4). The chemical stability of SMOP in the electrolyte was examined by immersing SMOP in a 1:1 (*v/v*) mixture of dioxolane (DOL) and dimethoxyethane (DME), followed by ^1^H NMR analysis after recovery and digestion. (Figure S9) The postimmersion spectrum retained the characteristic linker resonances of pristine SMOP without noticeable new decomposition‐related signals, indicating that SMOP remained chemically intact under the tested conditions. For the shuttle‐current measurement, which probes how actively Li‐PS species form and shuttle [51], cells were first galvanostatically discharged to 1.7 V and subsequently charged to 70% of the discharge capacity (70% of state of charge, SOC). After the open‐circuit voltage relaxed to a quasi‐steady value at 70% SOC, chronoamperometry was carried out at this equilibrium potential to monitor the Li‐PS shuttle current. As shown in Figure 4a, the two HCS‐based positive electrodes exhibit markedly lower shuttle currents than the KB‐based electrodes. SMOP‐S‐HCS shows the smallest steady‐state current (∼1‐2 µA at long times), whereas S‐HCS displays higher than SMOP‐S‐HCS but still lower than either S‐KB or SMOP‐S‐KB cells. The capacities associated with the shuttle current (*Q_ps_*), estimated by integrating the current‐time curves, are 76.9, 54.0, 96.7, and 92.6 mAh g_S_^−1^ for S‐HCS, SMOP‐S‐HCS, S‐KB, and SMOP‐S‐KB, respectively (Figure 4a and inset Figure). Because *Q_ps_* reflects the parasitic charge passed by Li‐PS shuttling, the lowest value for SMOP‐S‐HCS indicates the most effective suppression of Li‐PS shuttle. This behavior is consistent with a dual‐barrier architecture in SMOP‐S‐HCS, where sulfur confinement within the HCS cavity limits polysulfide release, and the outer SMOP coating provides polar binding sites that further immobilize Li‐PS species. In contrast, the KB‐based electrodes lack a defined core‐shell configuration and therefore exhibit substantially stronger shuttling. Notably, despite having the same SMOP loading, SMOP‐S‐KB shows only a modest reduction in shuttle relative to S‐KB, underscoring that SMOP placement, rather than its mere presence, is the dominant factor governing shuttle mitigation.

CV profiles (Figure 4b–e) for all four positive electrodes reveal the characteristic two discharge peaks and one charge peak associated with the stepwise reduction and reoxidation of sulfur [52]. A consistent host‐dependent effect of SMOP is observed. In the HCS‐based electrodes, SMOP‐S‐HCS shows sharper, better‐resolved redox peaks and a smaller cathodic‐anodic peak separation than S‐HCS, indicative of reduced polarization and improved reaction reversibility enabled by the conformal SMOP coating. In the KB‐based electrodes, SMOP‐S‐KB exhibits a similar but more modest improvement relative to S‐KB, consistent with the less controlled SMOP distribution. With increasing scan rate (0.05–0.3 mV s^−1^), all electrodes show the expected peak broadening and potential shifts; however, these changes are least pronounced for SMOP‐S‐HCS, suggesting more stable interfacial kinetics when SMOP is positioned as an outer barrier layer on HCS. Nyquist plots obtained after the CV measurement (Figure 4f–i) further highlight the roles of SMOP and the carbon host in governing interfacial resistance. All cells display a depressed semicircle in the high‐to‐medium frequency region, followed by an inclined low‐frequency tail attributable to diffusion‐limited processes. The semicircle diameter decreases upon SMOP incorporation for both carbon hosts, indicating reduced interfacial/charge‐transfer resistance, with SMOP‐S‐HCS exhibiting the smallest semicircle among the four cells. Together with the shuttle‐current results, these data suggest that the SMOP coating on HCS not only suppresses Li‐PS transport but also promotes more favorable interfacial charge transfer, likely by introducing polar, chemically anchoring sites at the cathode‐electrolyte interface while maintaining electronic continuity through the carbon framework [53].

**FIGURE 5:**
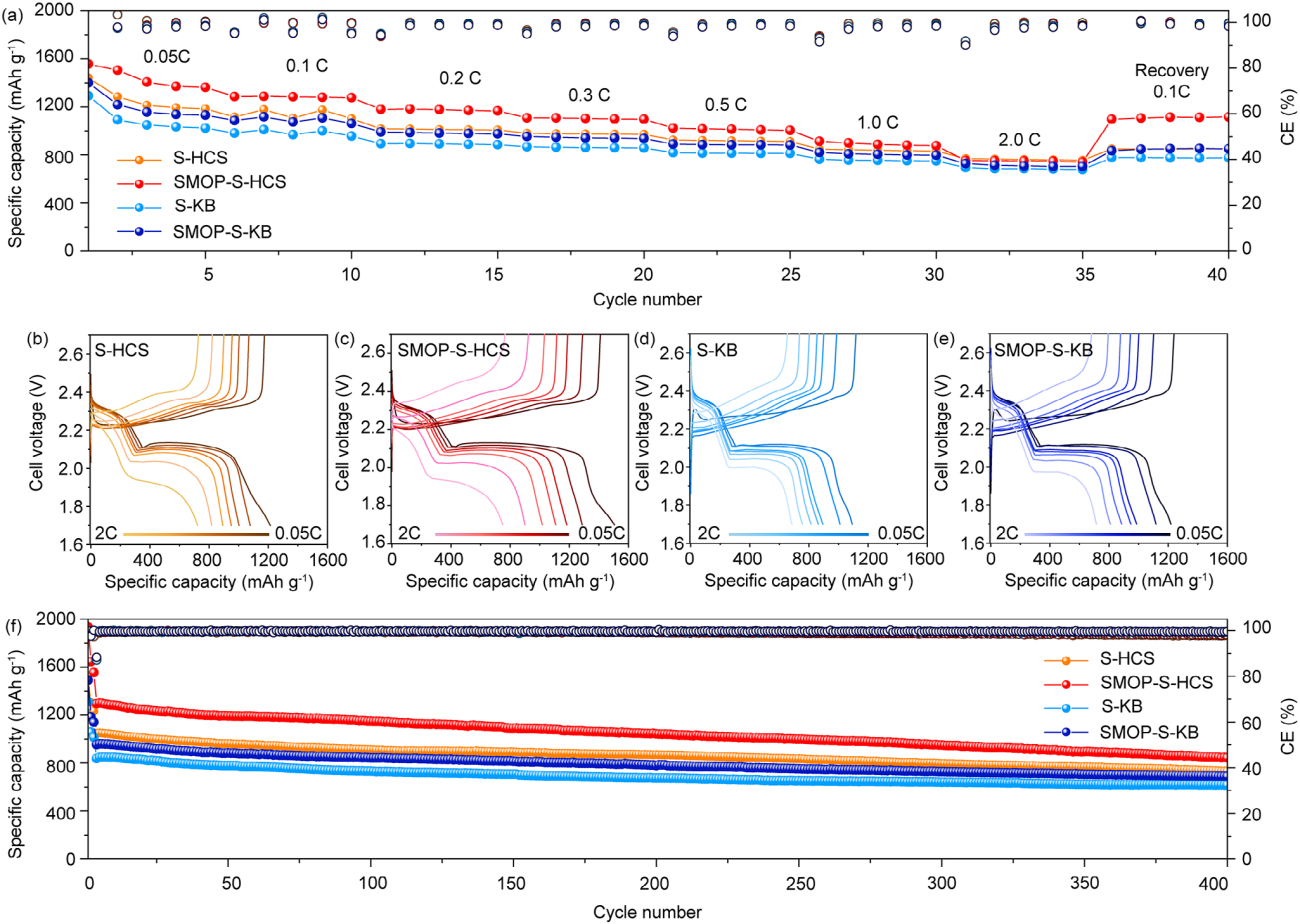
Electrochemical performance of S‐HCS, SMOP‐S‐HCS, S‐KB, and SMOP‐S‐KB positive electrodes. (a) Rate capability measured at C‐rates from 0.05 to 2.0C. Representative galvanostatic voltage profiles of (b) S‐HCS, (c) SMOP‐S‐HCS, (d) S‐KB, and (e) SMOP‐S‐KB positive electrodes collected at selected C‐rates. (e) Long‐term galvanostatic charge–discharge cycling performance at 0.3C. Areal sulfur loading of the electrodes is 1.5 mg_S_ cm^−2^.

Building on the interfacial/kinetic insights obtained from the shuttle‐current, CV, and EIS analyses, we next evaluated the practical electrochemical performance of the four electrodes (Figure 5). The rate‐capability results (Figure 5a) show that the SMOP‐S‐HCS positive electrode delivers the most robust capacity retention over the tested C‐rate range, decreasing from ∼1500 mAh g^−1^ at 0.05C to ∼800 mAh g^−1^ at 2.0C. Representative voltage profiles (Figure 5b–e) exhibit the characteristic two‐step discharge behavior, with two plateaus associated with sulfur reduction to soluble polysulfides followed by conversion to Li_2_S/Li_2_S_2_, in agreement with the CV results. As the C‐rate increases, voltage polarization becomes more pronounced for all electrodes, evidenced by a downward shift of the discharge plateaus and a reduction in plateau duration. This effect is slightly more apparent for the HCS‐based electrodes, likely because a larger fraction of sulfur is confined within the hollow spheres, which can impose additional mass‐transport and conversion‐kinetic constraints at the highest rate (2.0C). Importantly, this high‐rate limitation is largely reversible for SMOP‐S‐HCS positive electrodes. Upon returning to 0.1C, SMOP‐S‐HCS restores a prolonged discharge plateau and delivers the highest discharge capacity (1097.5 mAh g^−1^), exceeding those of S‐HCS (849.5 mAh g^−1^), S‐KB (777.8 mAh g^−1^), and SMOP‐S‐KB (834.4 mAh g^−1^) positive electrodes. This strong capacity recovery indicates that the reduced performance at 2.0C is primarily kinetic rather than due to permanent structural degradation. The superior rate performance and recovery are consistent with the synergistic host design in SMOP‐S‐HCS, where sulfur confinement within the HCS interior is coupled with polar SMOP sites that suppress polysulfide transport while maintaining efficient ion/electron transport. Coulombic efficiencies remain near unity throughout the rate test, suggesting limited parasitic loss during these short segments.

Long‐term cycling at 0.3C (Figure 5f) further demonstrates the advantage of SMOP‐S‐HCS, which delivers ∼1300 mAh g^−1^ in the first cycle and retains ∼850 mAh g^−1^ after 400 cycles. This performance surpasses that of S‐HCS (∼740 mAh g^−1^), S‐KB (∼620 mAh g^−1^), and SMOP‐S‐KB (∼690 mAh g^−1^) at the same cycle number. Consistently, the corresponding voltage–capacity profiles collected during cycling (Figure S10) show that SMOP‐S‐HCS better preserves the characteristic S_8_ to Li_2_S conversion plateaus around 2.1 V from the 1st to the 300th cycle with a comparatively smaller increase in voltage hysteresis, indicating more reversible sulfur utilization. Additional testing under more demanding conditions is provided in the Supporting Information, including high‐rate cycling data for the S‐HCS and SMOP‐S‐HCS electrodes at 1.0C with a sulfur loading of 1.5 mg_S_ cm^−2^, as well as representative galvanostatic voltage profiles at a higher sulfur loading of 3.0 mg_S_ cm^−2^ at 0.2C (Figures S11 and S12). These results show that the SMOP‐S‐HCS electrode retains its electrochemical advantage over the S‐HCS electrode even under elevated rate and higher‐loading conditions. Furthermore, to verify that the selected SMOP content was appropriate, we additionally evaluated SMOP‐S‐HCS electrodes containing 3, 6, and 9 wt.% SMOP (Figure S13). Among these, 3 wt.% SMOP delivered the best overall performance, whereas higher SMOP contents led to lower discharge capacities. Photographic (Figure S14) and SEM images (Figures S15–S17) showed progressively greater surface coverage with increasing SMOP content, while no micron‐scale SMOP crystals were observed, indicating that SMOP remained dispersed rather than recrystallizing. At the same time, electrodes containing 6 wt.% SMOP and above exhibited visible crack formation, suggesting that excessive additive loading compromised both transport and mechanical integrity. Accordingly, 3 wt.% SMOP was confirmed as the optimal loading for the main electrochemical comparisons.

**FIGURE 6:**
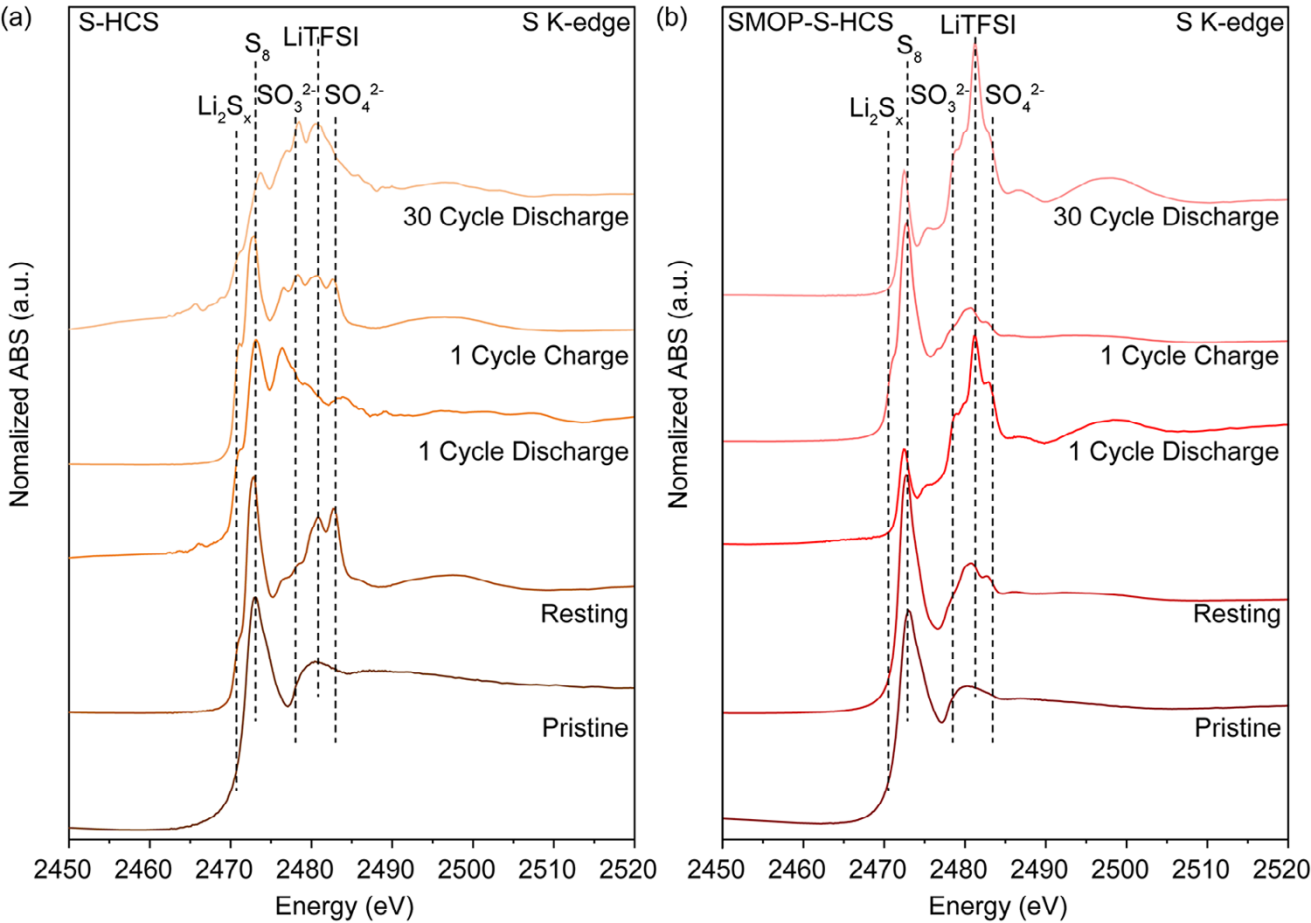
Ex situ S K‐edge XAS spectra of (a) S‐HCS and (b) SMOP‐S‐HCS collected at selected electrochemical states: pristine, after resting in electrolyte, after the first discharge, after the first charge, and after the 30th discharge. Electrodes were cycled at 0.1C prior to recovery for XAS. Areal sulfur loading of the electrodes is 1.5 mg_S_ cm^−2^.

Because HCS‐based architecture yields clearer performance gains than the KB‐based composites, we next probed how SMOP placement within the S‐HCS particles influences sulfur redox chemistry using sulfur K‐edge X‐ray absorption spectroscopy (XAS). Figure 6 presents ex situ sulfur K‐edge spectra of S‐HCS (Figure 6a) and SMOP‐S‐HCS (Figure 6b) collected at selected electrochemical states, (1) pristine electrodes, (2) pristine electrodes immersed in liquid electrolyte (“resting”), and electrodes retrieved after (3) the first discharge, (4) first charge, and (5) the 30th discharge at 0.1C‐rate. For XAS measurements, electrode coatings were harvested by scraping the electrode film from the current collector, and spectra were acquired in fluorescence mode.

For both pristine and resting samples, a dominant S_8_ feature is observed near ∼2472 eV. Weaker intensity at higher energies (∼2478–2484 eV) is also present, where contributions from the electrolyte salt (lithium bis(trifluoromethanesulfonyl)imide, LiTFSI) [54, 55] and SO_x_ species overlap [56, 57]. For SMOP‐S‐HCS, this region may additionally include contributions from SMOP sulfonate moieties. Upon the first discharge, the S_8_ feature decreases for both electrodes, consistent with sulfur reduction. Notably, S‐HCS develops a more pronounced lower‐energy feature (∼2468–2470 eV), assigned to reduced sulfur species (Li_2_S_x_/Li_2_S_2_/Li_2_S), whereas this feature is comparatively attenuated in SMOP‐S‐HCS. Importantly, this attenuated feature does not indicate suppressed sulfur reduction in SMOP‐S‐HCS, which is consistent with its electrochemical behavior, where SMOP‐S‐HCS positive electrodes deliver a higher discharge capacity than S‐HCS. Rather, the contrast is more consistent with discharge‐induced redistribution of sulfur species. In SMOP‐S‐HCS, reduced sulfur products are preferentially retained within the HCS interior and/or at internal SMOP‐associated interfaces, whereas in S‐HCS they more readily migrate into electrolyte‐accessible interparticle regions and external surfaces. As a result, their contribution becomes more prominent in the fluorescence XAS response of the scraped composite. This interpretation aligns with the role of the SMOP‐enriched outer layer in limiting outward transport of soluble intermediates and conversion products, thereby reducing their accumulation in electrolyte‐accessible particle‐to‐particle domains.

After the subsequent charge, the SMOP‐S‐HCS positive electrode largely restores an S_8_‐dominated profile with a strongly attenuated low‐energy feature, indicating more complete recovery of sulfur speciation. In contrast, the S‐HCS positive electrode retains a more noticeable low‐energy shoulder together with enhanced high‐energy S‐O_x_‐related contributions after charging, suggesting less complete speciation recovery and/or greater accumulation of irreversible sulfur‐containing byproducts [58, 59]. After 30 cycles, the S‐HCS spectrum becomes broader with diminished S_8_ definition and increased high‐energy contributions, whereas SMOP‐S‐HCS maintains comparatively better‐resolved sulfur features with less additional broadening, implying more reversible sulfur speciation and reduced buildup of irreversible components over extended cycling.

These ex situ S K‐edge XAS results provide mechanistic support for the performance advantages of SMOP‐S‐HCS. The HCS host confines sulfur within the hollow interior to limit outward migration of soluble intermediates, while the SMOP‐enriched outer surface serves as a chemically selective barrier that anchors polysulfides and stabilizes the cathode–electrolyte interface. This spatially integrated design is consistent with the suppressed shuttle current, reduced polarization, improved rate response, and markedly enhanced cycling stability observed for SMOP‐S‐HCS composite positive electrodes. In line with mitigation of parasitic shuttle‐mediated reactions, the fluorescence‐mode XAS spectra of scraped electrode composites indicate a more reversible evolution of sulfur speciation upon charge and a reduced buildup of oxidized sulfur‐related components over extended cycling. In addition, postcycling SEM‐EDS characterization of SMOP‐S‐HCS after 100 cycles at 0.1C (Figure S18) still showed HCS‐derived spherical features and detectable Zr signals, which do not support complete collapse or loss of the SMOP‐derived interfacial component during cycling. The comparison with the KB‐based composites further underscores that the impact of SMOP is governed by controlled interfacial deployment rather than additive loading alone; a conformal, functionally positioned layer translates chemical affinity into effective polysulfide regulation, whereas a dispersed/buried distribution provides only limited benefit.

## 3 Conclusions

In this study, SMOP are established as molecular‐scale interfacial modifiers for Li‐S sulfur positive electrodes. By intentionally deploying SMOP either as a conformal coating on sulfur‐loaded HCS or as a co‐deposited/physically blended additive in KB composites, the work clarifies that performance gains are dictated by interfacial presentation rather than additive identity or loading. When SMOP is positioned as an outer interfacial layer on the confinement‐type host, polysulfide shuttling is strongly suppressed, and interfacial kinetics are improved, leading to durable rate and cycling performance. In contrast, when SMOP is buried within aggregate networks without a defined surface layer, shuttle mitigation remains limited despite identical SMOP content. Ex situ sulfur K‐edge XAS further supports this design principle by showing more reversible sulfur speciation and reduced accumulation of oxidized sulfur‐related components in the SMOP‐coated HCS architecture during extended cycling. These findings provide a practical design rule for Li‐S electrodes, where the effective polysulfide regulation requires spatially controlled placement of functional sites at the active interface, not simply the introduction of polar additives. Discrete MOP cages offer a unique combination of molecular dispersibility and high functional‐group density, enabling ultrathin, high‐efficiency interfacial barriers at low inactive mass. Extending this approach to other cage chemistries, linkers, and host morphologies is expected to broaden the scope of molecularly engineered interfaces for stabilizing sulfur electrodes.

## 4 Methods

### 4.1 Synthesis of SMOP Crystal and SMOP Unit

SMOP crystal was synthesized by dissolving Cp_2_ZrCl_2_ (225 mg, 0.77 mmol, Thermo Fisher Scientific), terephthalic acid (93.44 mg, 0.56 mmol, Thermo Fisher), and monosodium 2‐sulfoterephthalate (50.28 mg, 0.19 mmol, Tokyo Chemical Industry) in a solvent mixture of N,N‐dimethylformamide (DMF, 6.25 mL, Thermo Fisher, 99.7%), deionized (DI) water (1.125 mL), and tetrahydrofuran (THF, 7.5 mL, Thermo Scientific, 99.7%). The solution was placed in a 20 mL glass vial and reacted in an oven at room temperature (∼23°C) for 16 h. The resulting white precipitate in the solution was collected and washed 3 times with a mixture of DMF and THF (1:1 v/v, 10 mL) by centrifugation to remove unreacted precursors. To obtain the discrete SMOP cage, the SMOP crystal was socked in methanol for 3 days, during which the crystals dissociated into molecular cages. The suspension was then centrifuged, and the solid was recovered by freeze‐drying.

### 4.2 Synthesis of S‐HCS, SMOP‐S‐HCS, S‐KB, and SMOP‐S‐KB

To prepare S‐HCS composite, the 30 mg HCS (ACS Material) was dispersed in the mixture solution of IPA (Thermo Fisher, technical grade) and CS_2_ (Thermo Scientific Chemicals, 99.9%) at a volume ratio of 2:3, containing 170 mg of dissolved elemental sulfur (SkySpring Nanomaterials, Inc.) [39]. The suspension was heated to 50°C with mild stirring for 16 h to evaporate the solvents. The resulting black powder (S‐HCS) was further dried in a vacuum oven at 50°C overnight. According to thermogravimetric analysis (TGA), the sulfur content of the as‐prepared S‐HCS composite is approximately 78 wt.%. For the preparation of SMOP‐S‐HCS, SMOP was introduced via solvent evaporation after sulfur infiltration. Specifically, 10 mg of SMOP powder was added to the S‐HCS suspension in IPA, followed by solvent evaporation and drying under the same conditions as used for the S‐HCS composite. The mass ratio of sulfur–carbon composite to SMOP was fixed at 97:3 (w/w), corresponding to 3 wt.% SMOP in the final composite. Accordingly, the sulfur content in SMOP‐S‐HCS is approximately 75.7 wt.%, assuming quantitative SMOP retention and unchanged sulfur loading. For comparison, samples containing higher SMOP loadings of 6 and 9 wt.% were also prepared by adding 20 mg and 30 mg of SMOP, respectively, to the S‐HCS suspension in IPA, followed by the identical solvent evaporation and drying process, and were subsequently subjected to SEM characterization and electrochemical testing.

For the S‐KB composite, 800 mg of elemental sulfur is dissolved in mixture solution of CS_2_ and N‐Methyl‐2‐pyrrolidone (NMP, Sigma Aldrich, 99%) at volume ratio of 2:3. Then, the 200 mg of KB was added to the sulfur solution and thoroughly mixed using a mortar and pestle to promote solvent evaporation and to distribute sulfur elemental sulfur uniformly on the KB matrix [55]. The obtained black powder (S‐KB) was further dried in a vacuum oven at 50°C overnight. TGA analysis indicates that the sulfur content of the S‐KB composite is approximately 78 wt.%. SMOP‐S‐KB was prepared by adding 30 mg of SMOP powder to the S‐KB suspension, followed by mixing and drying using the same procedure as for the S‐KB composite. The sulfur–carbon composite to SMOP mass ratio was maintained at 97:3 (w/w), yielding a final sulfur content of approximately 75.7 wt.% in the SMOP‐S‐KB composite.

### 4.3 Material Characterization

The morphology and elemental distribution of the samples were examined by scanning electron microscopy (SEM, Auriga, Zeiss) equipped with energy‐dispersive X‐ray spectroscopy (EDS, Oxford Instruments). Transmission electron microscopy (TEM, ARM200F and JEM‐2100F, JEOL) and Cs‐corrected scanning transmission electron microscopy (STEM) coupled with EDS (X‐Max, Oxford Instruments) were used to further investigate the morphology and elemental distribution. For SEM observations, powder samples were mounted on aluminum stubs using conductive carbon tape to ensure proper adhesion and minimize electron charging, and images were acquired at an acceleration voltage of 5 kV. Powder X‐ray diffraction patterns were collected using a Bruker D8 Advance (TRIO/TWIN) diffractometer operated at 40 kV and 40 mA (1600 W). Data were recorded using Cu Kα radiation over a 2θ range of 5°–60° at a scan rate of 4°min^−1^, with samples mounted on a silicon sample holder. ^1^H spectra of digested SMOPs were recorded on a Bruker Avance III HD 500 MHz spectrometer. For NMR, ∼3 mg of dried SMOP was digested and dissolved in a mixture of DMSO‐d_6_ and Deuterium chloride (dcl) under sonication. The chemical stability of the synthesized SMOP was evaluated by soaking the powder in a DOL/DME solvent mixture (1:1 v/v) at a solid‐to‐solvent ratio of 1 mg mL^−1^ inside a glovebox for 8 h. After soaking, the solid was recovered by centrifugation and dried overnight in a fume hood. The recovered sample was then digested in DMSO‐d_6_/DCl using the same procedure described above prior to NMR analysis. TGA was conducted using a LABSYS EVO (Setaram) under a He flow. Samples were heated from room temperature to 100°C at 20°C min^−1^, held for 10 min, heated to 800°C at 5°C min^−1^, followed by cooling to 50°C at 20°C min^−1^.

XPS of sulfur‐carbon composites was conducted using an Axis Supra+ spectrometer (Kratos Analytical Ltd., Manchester, UK) with a monochromatic Al Kα radiation source. All XPS spectra are normalized to the maximum peak intensity for ease of comparison. N_2_ adsorption–desorption isotherms were measured at 77 K using the Tristar II Plus (Micromeritics) gas adsorption analyzer. Samples were prepared and measured after evacuating at 50°C for 24 h. Raman spectra were collected using a custom‐built multi‐wavelength Raman spectrometer under ambient conditions. The spectra were acquired using an Acton 300i spectrograph, and the sample was excited with a 532 nm Coherent Sapphire SF laser operating at a maximum output power of 150 mW.

### 4.4 Electrochemical Tests

Sulfur‐carbon composite electrodes were prepared by preparing a slurry consisting of 60 wt.% of the synthesized sulfur‐carbon composites, 28.5 wt.% Super‐P (Timcal), 1.5 wt.% of Graphene oxide (Graphenea), and 10 wt.% of LA133 binder (MSE Supply) dissolved in DI water (Sulfur content in electrode is 46.8% for S‐HCS and S‐KB, 45.4% for SMOP‐S‐HCS and SMOP‐S‐KB). Thus, the content of SMOP in the SMOP‐containing electrodes is 1.8 wt.%. The mixture was blended using a centrifuge mixer (Thinky ARE‐310) for 1 h. The slurry was cast onto carbon‐coated aluminum foil (Wellcos) using a doctor blade. Different applicator gaps were employed to control the sulfur loading, yielding electrodes with areal sulfur loadings of ∼1.5 and ∼3.0 mg_S_ cm^−2^. The electrodes were dried at room temperature and subsequently vacuum‐dried at 50°C overnight. The dried electrodes were then punched into 12 mm diameter discs. 2032 coin‐type cells were assembled in an Ar‐filled glovebox (O_2_ and H_2_O < 0.1 ppm). The areal loading of electrodes is 1.5 mg_S_/cm^2^. Lithium metal (Honjo metal, 120 µm) as the counter electrode, and Celgard 2400 as a separator and the electrolyte consisting of 1 M LiTFSI (Sigma‐Aldrich, 99.99%) and 3 wt.% lithium nitrate (LiNO_3_, Strem, 99%) dissolved in a 1:1 (v/v) mixture of dioxolane (DOL, Sigma‐Aldrich, 99.5%) and dimethoxyethane (DME, Sigma‐Aldrich, 99.5%) were used.

Galvanostatic charge‐discharge cycling tests were performed using a battery cycling tester (Arbin Instrument) within the voltage window of 1.7–2.7 V. For postcycling SEM–EDS characterization, electrodes were harvested from cells that had been subjected to galvanostatic cycling at 1.0C for 100 cycles and collected at the fully charged state prior to disassembly in an argon‐filled glovebox. The electrodes were rinsed several times with anhydrous DME to remove residual electrolyte and soluble polysulfides and then dried under vacuum inside the glovebox. The dried electrodes were mounted on aluminum stubs using conductive carbon tape, and SEM–EDS characterization was performed at an accelerating voltage of 20 kV. EIS was performed using a Gamry Reference 3000 potentiostat/galvanostatic (Gamry Instruments, USA) over the frequency range of 1 MHz to 50 mHz using a 5‐mV stimulus. For shuttle‐current measurements, the assembled cells were initially fully discharged, followed by charging to 70% of their discharge capacity. After resting it for 24 h to measure the equilibrium voltage, the cells were potentiostatically charged at the measured equilibrium voltage for 40 h while the current was continuously recorded. A baseline background current was measured separately and subtracted from the recorded potentiostatic‐hold current to isolate the polysulfide shuttle contribution. The resulting shuttle current profile was used to quantify the parasitic redox reactions occurring during the hold. The total polysulfide shuttle capacity (*Q_ps_*) was obtained by integrating the baseline‐corrected current over the 40 h voltage‐holding period. CV was performed within the voltage window of 1.7 and 2.7 V at scan rates of 0.05, 0.1, and 0.3 mV s^−1^ to analyze the redox behavior of the sulfur‐based positive electrodes.

For postmortem XAS analysis, Li‐S cells were subjected to controlled electrochemical conditioning prior to disassembly. Cells were rested for 24 h, followed by galvanostatic charging or discharging at 0.1C to the target cutoff voltages. Charged samples were obtained by charging the cells to 2.7 V and subsequently holding the voltage for 10 h, whereas discharged samples were prepared by discharging the cells to 1.7 V followed by a 10 h voltage hold. After electrochemical conditioning, the cells were transferred to an argon‐filled glovebox and disassembled. The electrodes were gently rinsed several times with anhydrous DME to remove residual electrolyte and soluble polysulfides, followed by drying under vacuum inside the glovebox. The cleaned electrodes (pristine, resting, and cycled states) were sealed in air‐tight sample holders with X‐ray‐transparent windows to prevent exposure to air and moisture during measurement. Sulfur K‐edge XANES spectra were acquired in fluorescence mode at SSRL beamline 4–3, utilizing a Si(111) double‐crystal monochromator. X‐ray energy ranges from 2465 to 2484 eV near the S K‐edge energy (2472 eV) using a Passivated Implanted Planar Silicon (PIPS) fluorescence detector placed at a 45‐degree angle from the sample. Athena software from the Demeter package was used for processing X‐ray absorption near‐edge spectroscopy (XANES) data.

## Funding

New faculty start‐up fundings of Prof. Hwa and Prof. Mu at Arizona State University. National Research Foundation of Korea (NRF), funded by the Korea government (MSIT), Grant No. RS‐2022‐NR070845; Ministry of Trade, Industry and Energy (MOTIE, Korea), Industrial Technology Alchemist Project, Grant No. 20025773.

## Conflicts of Interest

The authors declare no conflicts of interest.

## Supporting information




**Supporting File**: advs75347‐sup‐0001‐SuppMat.docx.

## Data Availability

The data that support the findings of this study are available from the corresponding author upon reasonable request.
