# Supplementary material for: Interfacial Polysulfide Confinement via Spatially Controlled Sulfonated Metal–Organic Polyhedra Coatings in Lithium–Sulfur Batteries
Source: Adv Sci (Weinh). 2026 Apr 16;13(38):e75347. doi: 10.1002/advs.75347 (PMC13335555; doi:10.1002/advs.75347)
Supplement: Supplementary file 1 — Supporting File: advs75347‐sup‐0001‐SuppMat.docx. [file ADVS-13-e75347-s001.docx]

Supporting Information

Interfacial Polysulfide Confinement via Spatially Controlled Sulfonated Metal-Organic Polyhedra Coatings in Lithium–Sulfur Batteries

Soyeon Ko,^1^ WooYeon Moon,^2^ Yuwei Zhu,^3^ Yuhui An,^3^ Yunyuan Lu,^3^ Sourayan Bal,^3^ Linqin Mu,^3^ Kyung Min Choi,^2,*^ and Yoon Hwa^4,*^

S. Ko

^1^Chemical Engineering, Fulton Schools of Engineering,

Arizona State University,

Tempe, AZ 85287, USA

WY Moon, KM Choi

^2^Department of Chemical and Biological Engineering,

Sookmyung Women’s University,

100 Cheongpa-ro 47-gil, Yongsan-gu, Seoul 04310, Republic of Korea.

Email: [kmchoi@sookmyung.ac.kr](mailto:kmchoi@sookmyung.ac.kr) (K. Choi)

Y. Zhu, Y. An, Y. Lu, S. Bal, L. Mu

^3^Materials Science and Engineering, Fulton Schools of Engineering,

Arizona State University,

Tempe, AZ 85287, USA

Y. Hwa

^4^School of Electrical, Computer and Energy Engineering,

Arizona State University,

Tempe, AZ 85287, USA

Email: [Yoon.Hwa@asu.edu](mailto:Yoon.Hwa@asu.edu) (Y. Hwa)

Table of Contents

1. Characterization of carbon hosts 4

**Figure S1.** SEM image of hollow carbon sphere 4

**Figure S2.** SEM image of ketjen black 5

2. Characterizations of sulfur-carbon composites and SMOP 6

**Figure S3.** TGA results of (a) S-HCS and (b) S-KB under helium atmosphere with a ramping rate of 5 ^o^C min^-1^.. 6

**Figure S4.** Nitrogen adsorption (filled symbols) and desorption (open symbols) isotherms of KB and HCS measured at 77 K. Samples were degassed at 60 °C under vacuum/N_2_ for 15 h prior to measurement. 7

**Figure S5.** Nitrogen adsorption (filled symbols) and desorption (open symbols) isotherms of S-KB, SMOP-S-KB, S-HCS, and SMOP-S-HCS measured at 77 K Samples were degassed at 60 °C under vacuum/N_2_ for 15 h prior to measurement. …8

**Figure S6.** Pore size distribution analysis results of HCS and KB 9

**Figure S7.** Pore size distribution analysis results of S-HCS, SMOP-S-HCS, S-KB, and SMOP-S-KB 10

**Figure S8.** XPS survey spectra of SMOP, SMOP-S-HCS, and SMOP-S-KB showing the overall elemental composition of each sample... 11

**Figure S9.** ¹H NMR spectrum of SMOP after soaking in a DOL/DME mixture solvent (1:1 *v/v*) 12

**Table S1.** BET surface area of HCS, KB, S-HCS, SMOP-S-HCS, S-KB, and SMOP-S-KB composites. 13

3. Electrochemical characterization of S-C composite positive electrodes 14

**Figure S10.** Voltage profiles of (a) S-HCS, (b) SMOP-S-HCS, (c) S-KB, and (d) SMOP-S-KB positive electrodes during charge-discharge cycling test at 0.3 C, corresponding to Figure 5f. Areal sulfur loading of the electrodes is 1.5 mg_S_ cm^-2^.. 14

**Figure S11.** High-rate cycling performance of S-HCS and SMOP-S-HCS positive electrodes at 1.0 C with an areal sulfur loading of 1.5 mg_S_ cm^-2^. 15

**Figure S12.** Voltage profile of (a) S-HCS and (b) SMOP-S-HCS positive electrodes at 0.2 C with an areal sulfur loading of 3 mg_S_ cm^-2^. 16

**Figure S13.** Electrochemical performance of SMOP-S-HCS positive electrodes with varying SMOP contents. 17

**Figure S14.** Digital photographs of SMOP-S-HCS positive electrodes containing 3, 6, and 9 wt% SMOP. Surface irregularities become more apparent with increasing SMOP content, particularly at 6 and 9 wt%. 18

**Figure S15.** SEM images of the SMOP-S-HCS positive electrode containing 3 wt% SMOP at (a) low and (b) higher magnification. 19

**Figure S16.** SEM images of the SMOP-S-HCS positive electrode containing 6 wt% SMOP at (a) low and (b) higher magnification. 19

**Figure S17.** SEM images of the SMOP-S-HCS positive electrode containing 3 wt% SMOP at (a) low and (b) higher magnification. 20

**Figure S18.** Post-cycling SEM-EDS characterization of the SMOP-S-HCS positive electrode after 100 cycles at 0.1 C. Backscattered electron (BSE) image and corresponding elemental mapping results of C, O, S, and Zr. 20

**Characterization of SMOP (MOP-SO_3_H) and carbon hosts**


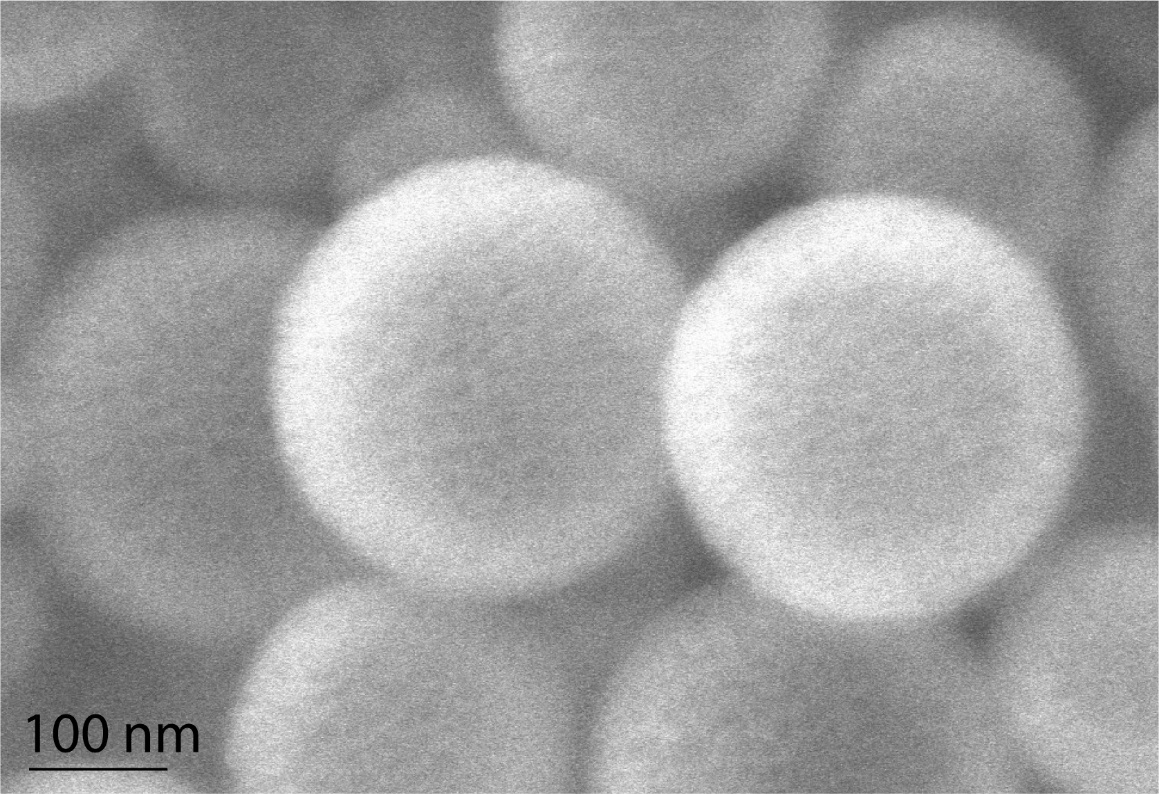


**Figure S1.** SEM image of hollow carbon sphere.


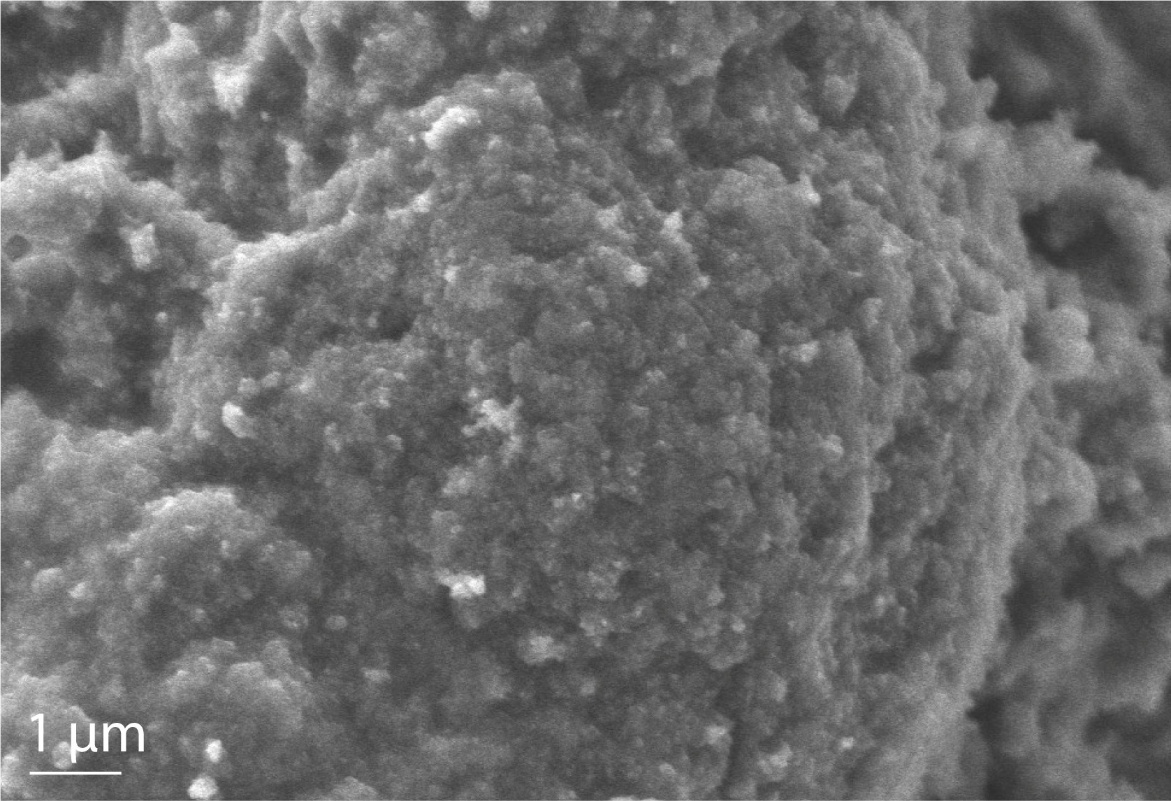


**Figure 2.** SEM image of ketjen black **2. Characterizations of sulfur-carbon composites (S-HCS, SMOP-S-HCS, S-KB, SMOP-S-KB) and SMOP**


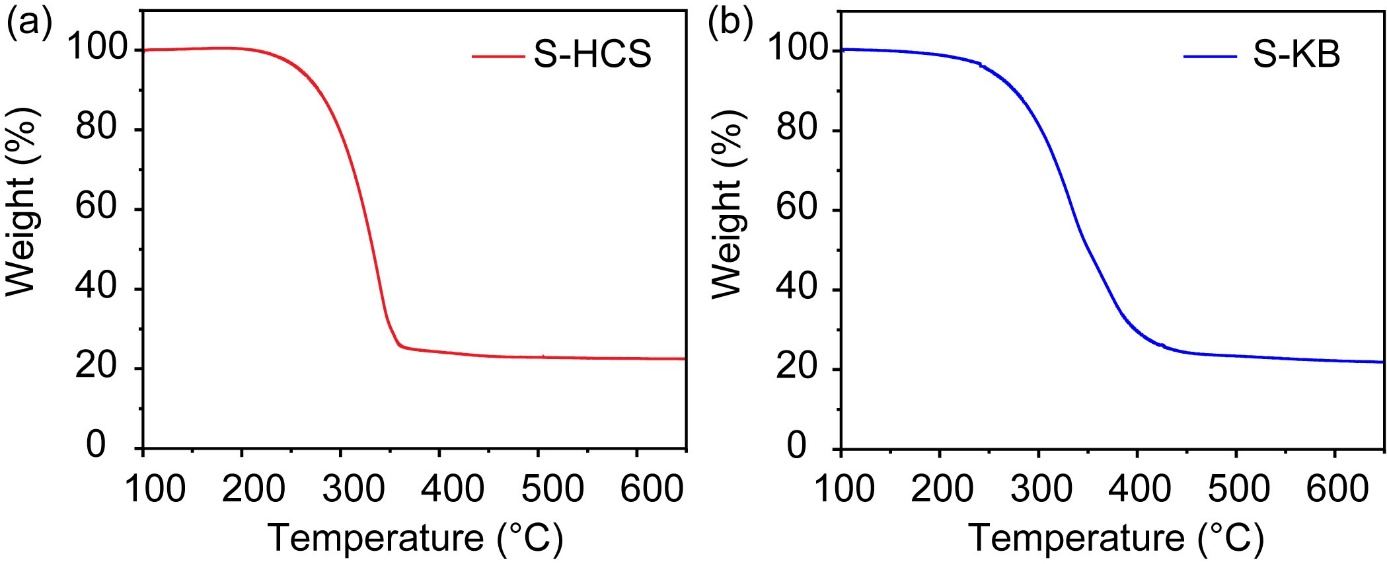


**Figure S3.** TGA results of (a) S-HCS and (b) S-KB under helium atmosphere with a ramping rate of 5 ^o^C min^-1^.

**

**

**Figure S4.** Nitrogen adsorption (filled symbols) and desorption (open symbols) isotherms of KB and HCS measured at 77 K. Samples were degassed at 60 °C under vacuum/N_2_ for 15 h prior to measurement.


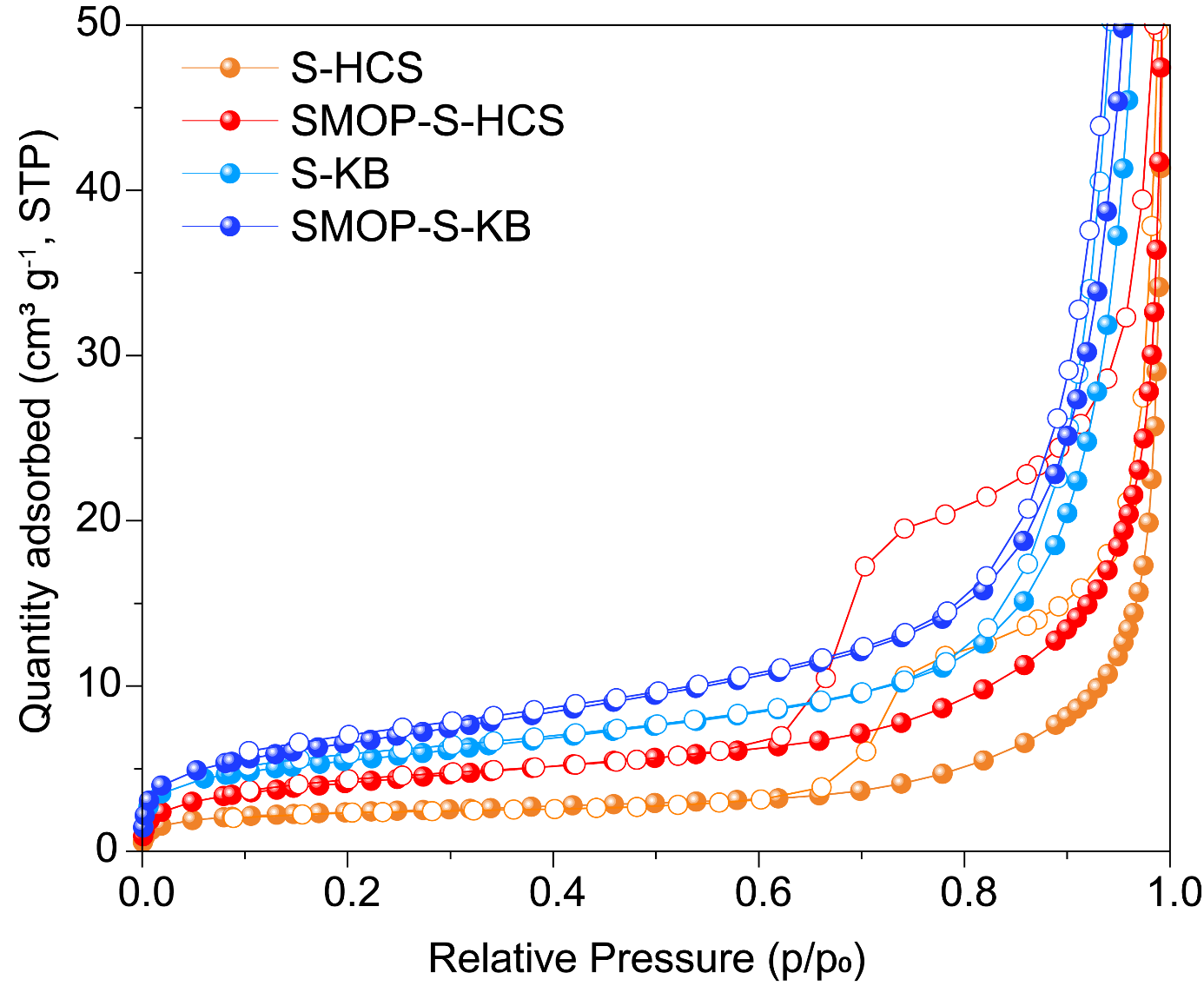


**Figure S5.** Nitrogen adsorption (filled symbols) and desorption (open symbols) isotherms of S-KB, SMOP-S-KB, S-HCS, and SMOP-S-HCS measured at 77 K Samples were degassed at 60 °C under vacuum/N_2_ for 15 h prior to measurement.

**
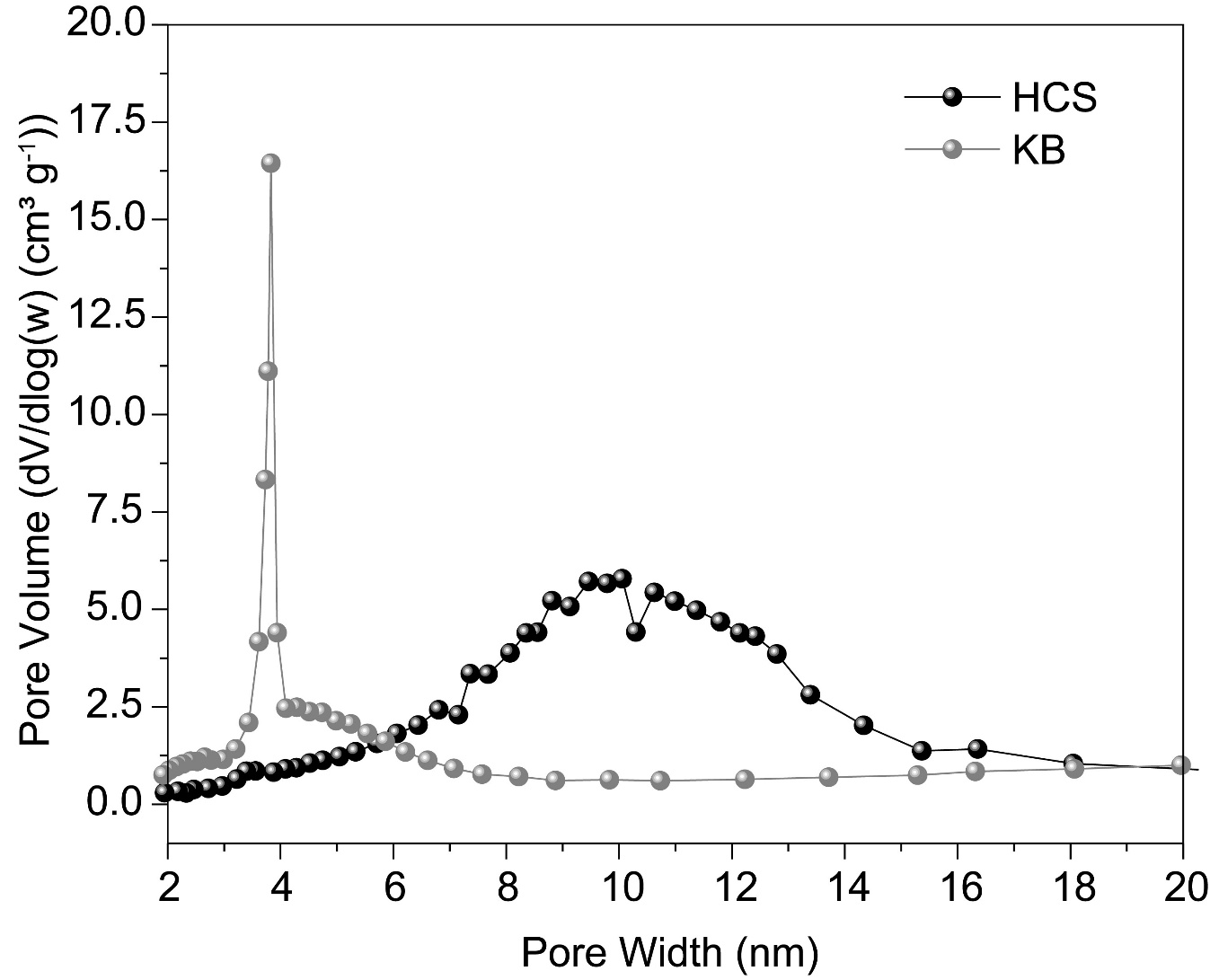
**

**Figure S6.** Pore size distribution analysis results of HCS and KB.

**
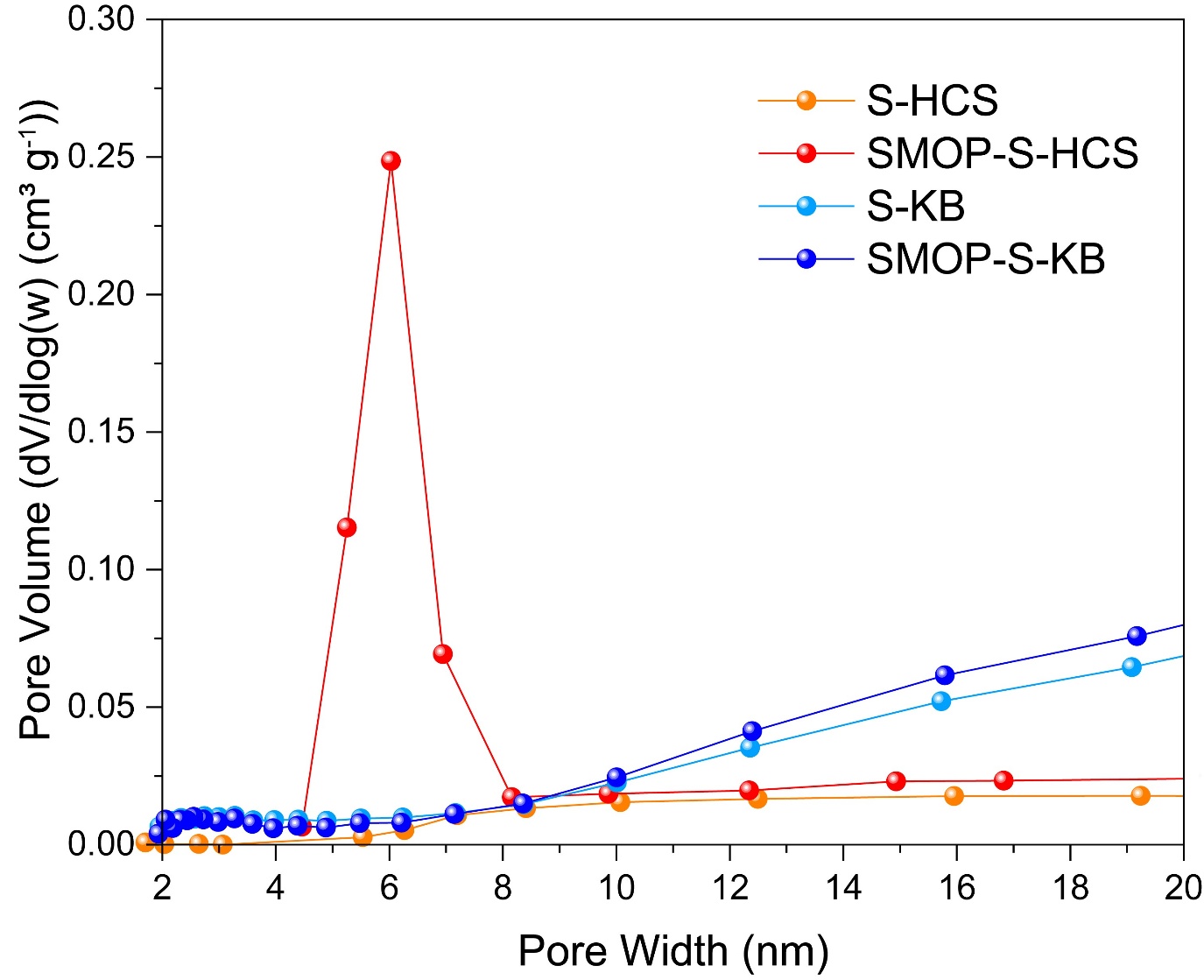
**

**Figure S7.** Pore size distribution analysis results of S-HCS, SMOP-S-HCS, S-KB, and SMOP-S-KB.

**
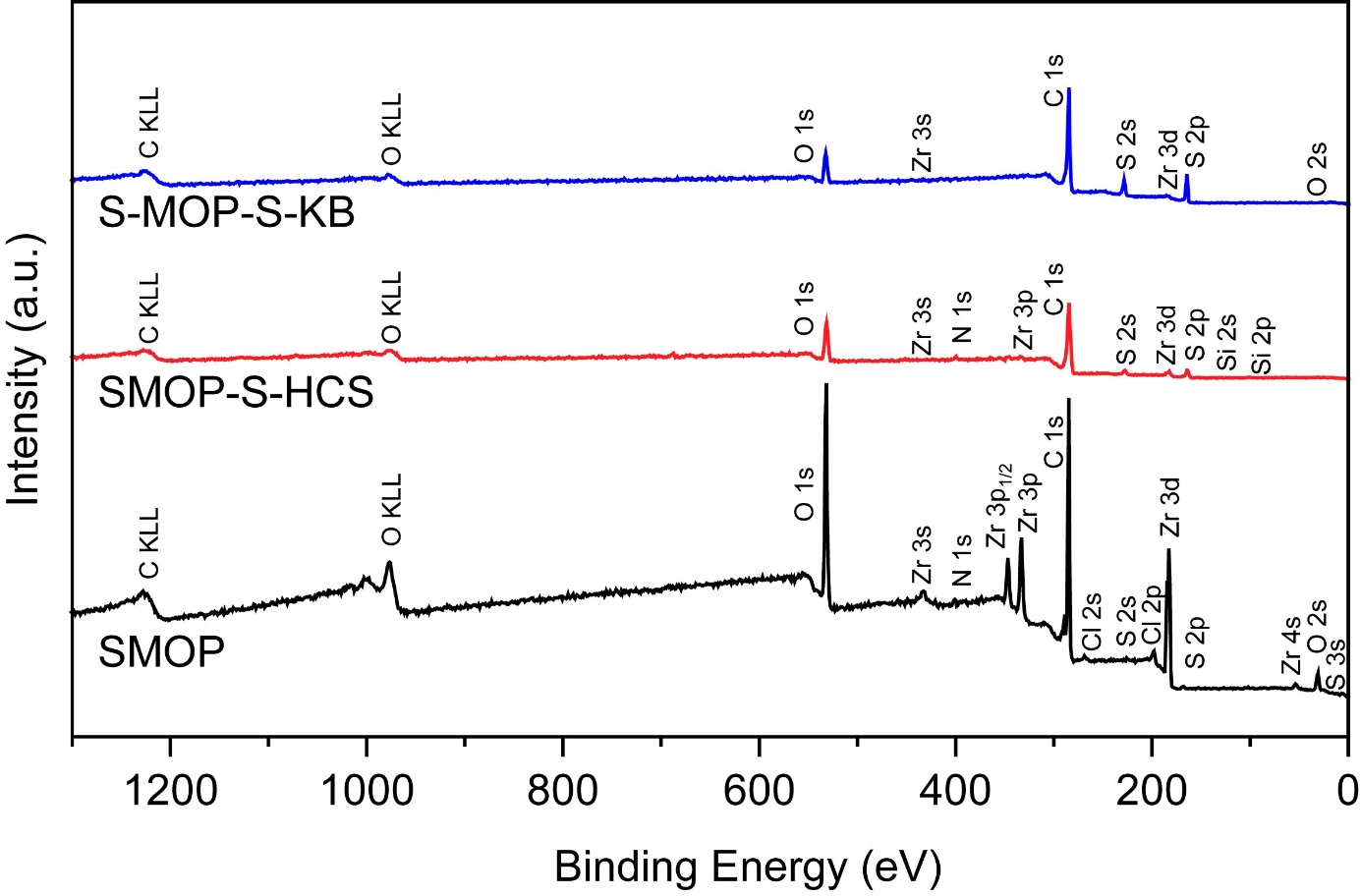
**

**Figure S8.** XPS survey spectra of SMOP, SMOP-S-HCS, and SMOP-S-KB showing the overall elemental composition of each sample.

**
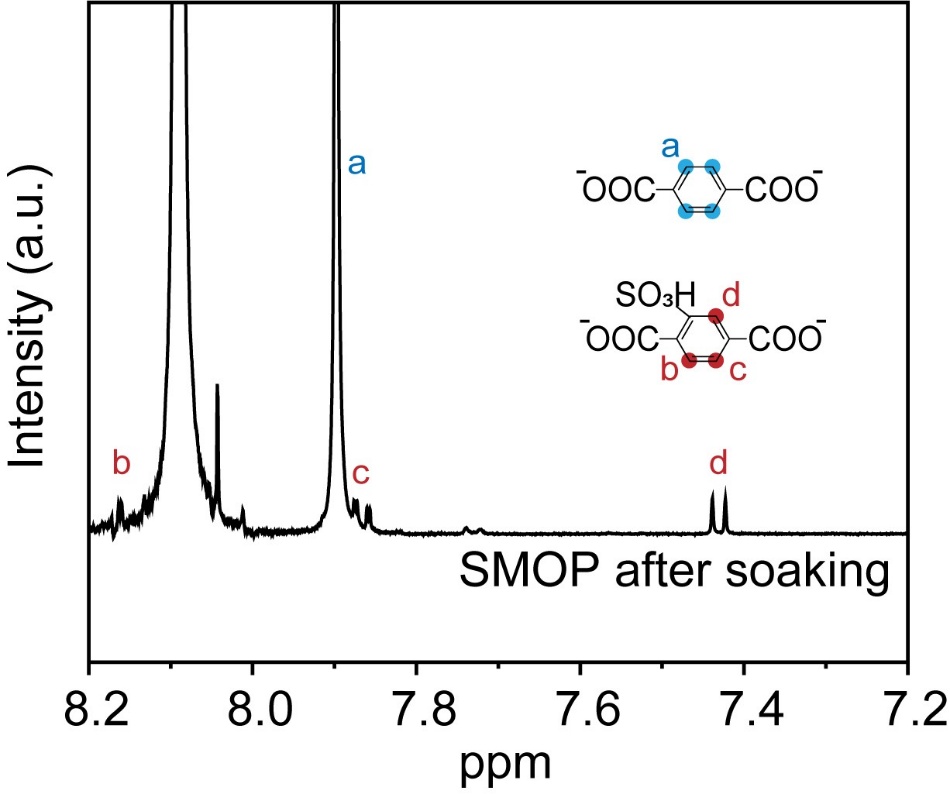
**

**Figure S9.** ¹H NMR spectrum of SMOP after soaking in a DOL/DME mixture solvent (1:1 *v/v*).

Table S1. BET surface area of HCS, KB, S-HCS, SMOP-S-HCS, S-KB, and SMOP-S-KB composites.

| Materials | BET Surface area (m^2^ g^−1^) |
| --- | --- |
| HCS | 1,378.44 |
| KB | 1,305.23 |
| S-HCS | 8.66 |
| SMOP-S-HCS | 14.94 |
| S-KB | 18.93 |
| SMOP-S-KB | 23.42 |

**3. Electrochemical characterization of S-C composite positive electrodes**


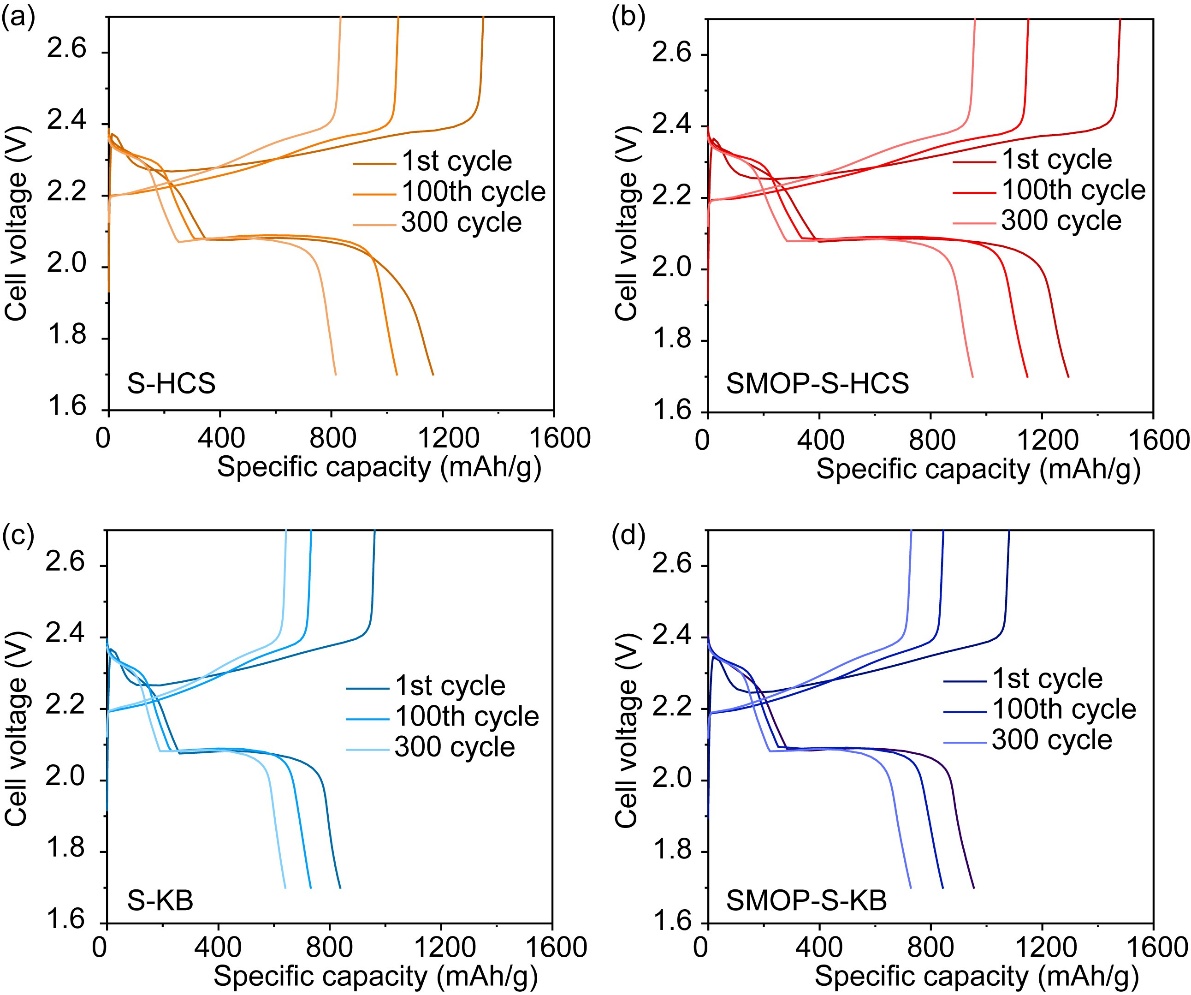


**Figure S10.** Voltage profiles of (a) S-HCS, (b) SMOP-S-HCS, (c) S-KB, and (d) SMOP-S-KB positive electrodes during charge-discharge cycling test at 0.3 C, corresponding to Figure 5f. Areal sulfur loading of the electrodes is 1.5 mg_S_ cm^-2^.

**
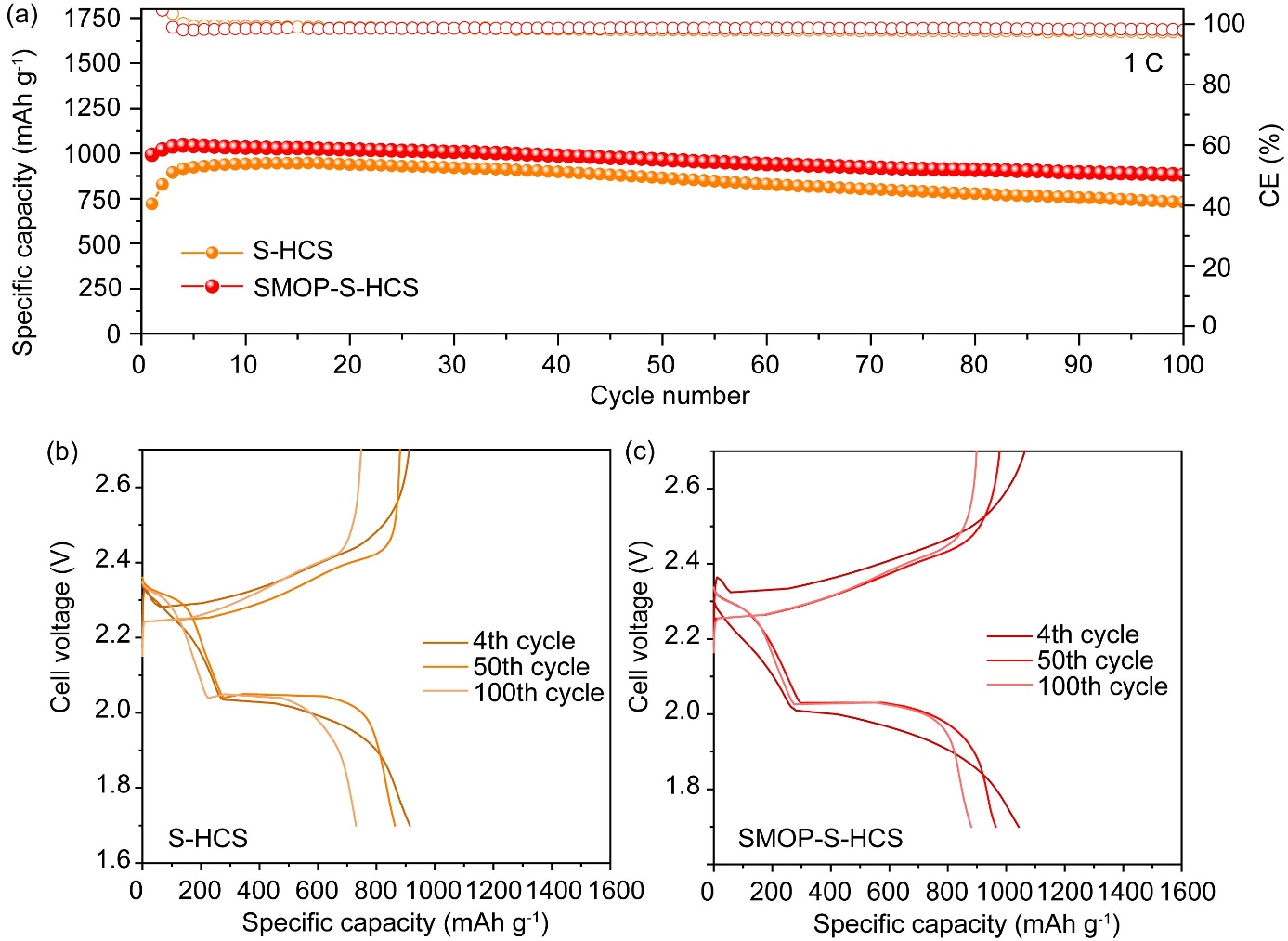
**

**Figure S11.** High-rate cycling performance of S-HCS and SMOP-S-HCS positive electrodes at 1.0 C with an areal sulfur loading of 1.5 mg_S_ cm^-2^. (a) Long-term galvanostatic charge-discharge cycling performance and Coulombic efficiency. Representative galvanostatic voltage profiles of (b) S-HCS and (c) SMOP-S-HCS electrodes collected at selected cycles (4th, 50th, and 100th cycles)

.

**
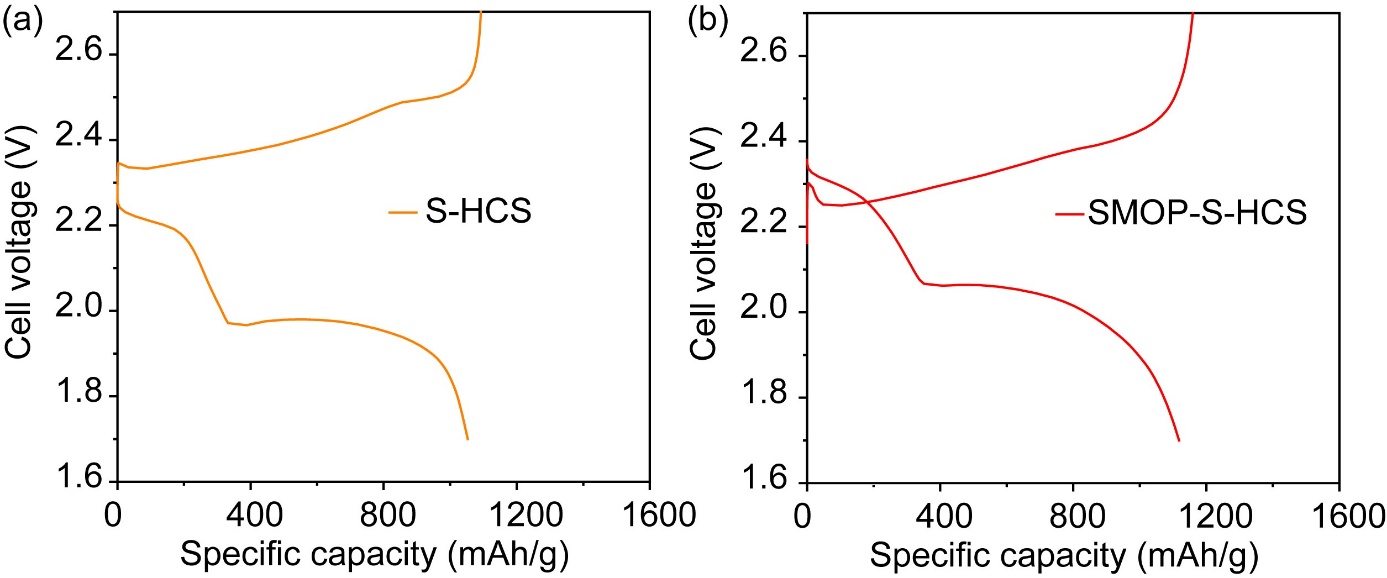
**

**Figure S12.** Voltage profile of (a) S-HCS and (b) SMOP-S-HCS positive electrodes at 0.2 C with an areal sulfur loading of 3 mg_S_ cm^-2^.

**
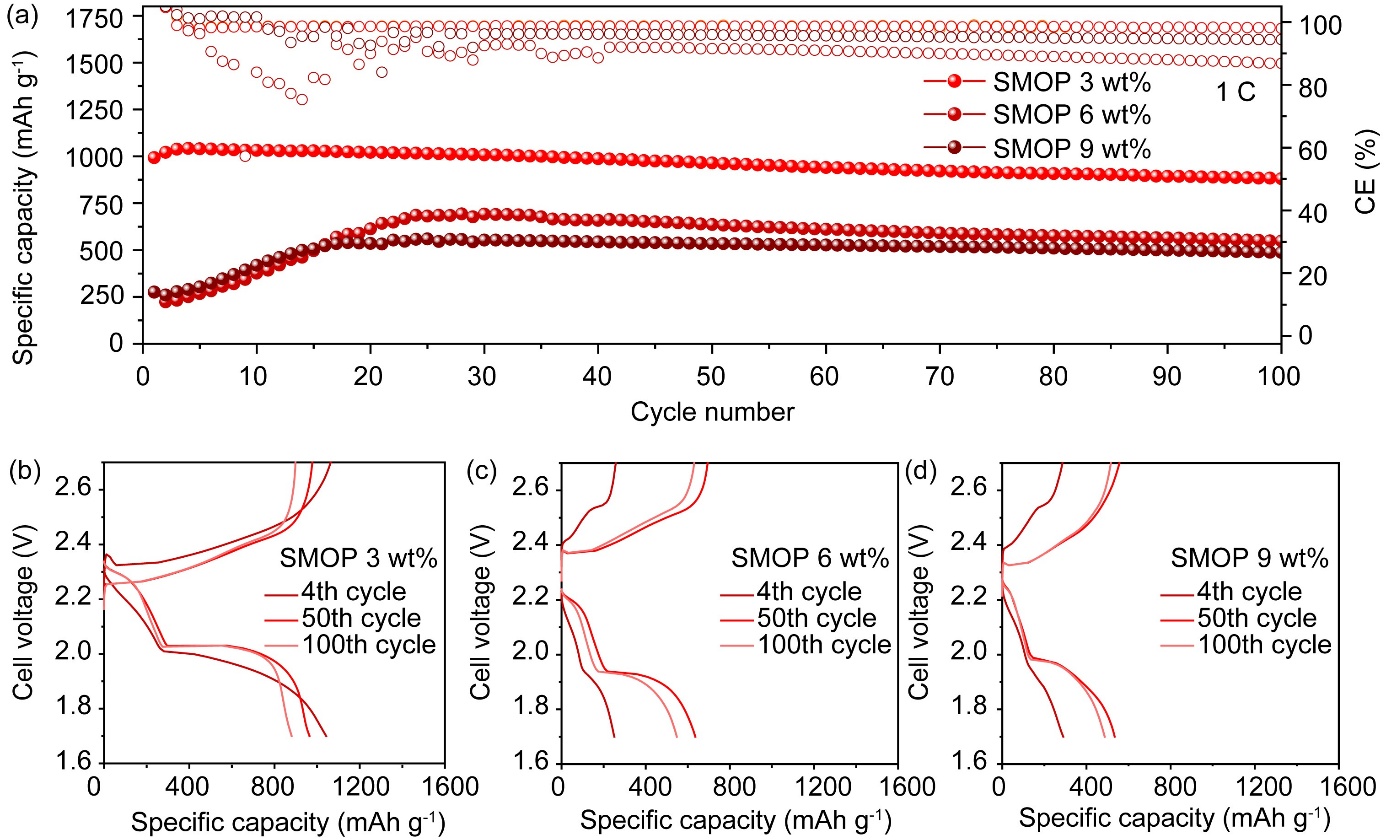
**

**Figure S13.** Electrochemical performance of SMOP-S-HCS positive electrodes with varying SMOP contents. (a) Long-term galvanostatic charge–discharge cycling performance at 1.0 C. Representative galvanostatic voltage profiles of SMOP-S-HCS electrodes containing (b) 3 wt%, (c) 6 wt%, and (d) 9 wt% SMOP in SMOP-S-HCS composite at selected cycles. Areal sulfur loading of the electrodes is 1.5 mg_S_ cm^-2^.

**
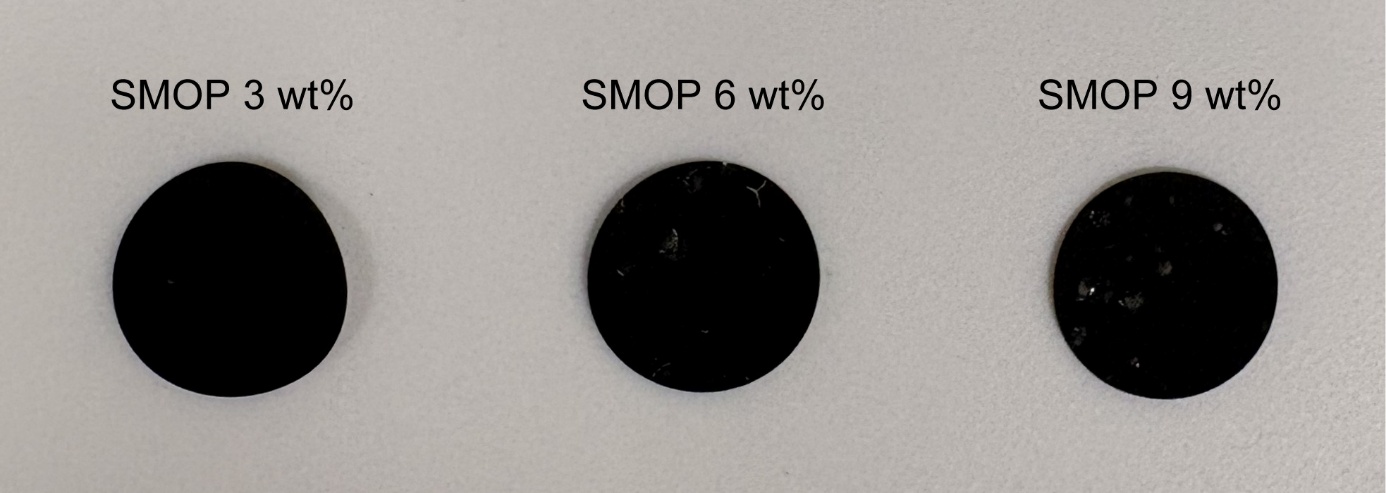
**

**Figure S14.** Digital photographs of SMOP-S-HCS positive electrodes containing 3, 6, and 9 wt% SMOP. Surface irregularities become more apparent with increasing SMOP content, particularly at 6 and 9 wt%.


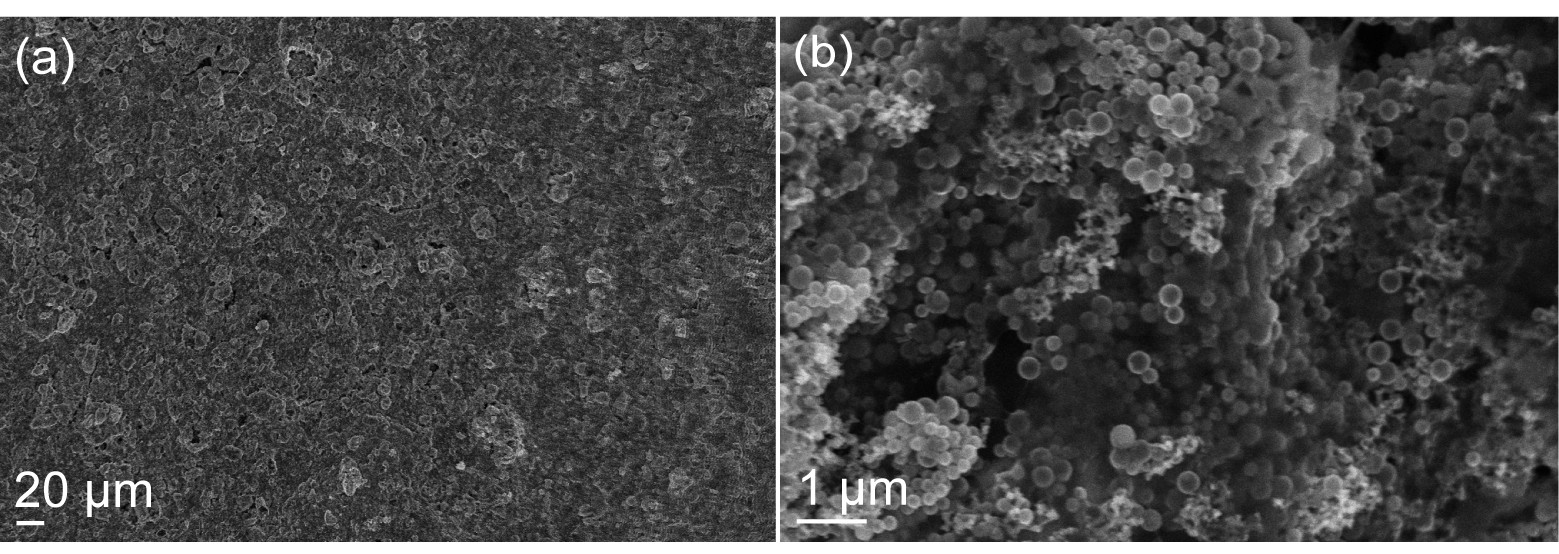


**Figure S15.** SEM images of the SMOP-S-HCS positive electrode containing 3 wt% SMOP at (a) low and (b) higher magnification.


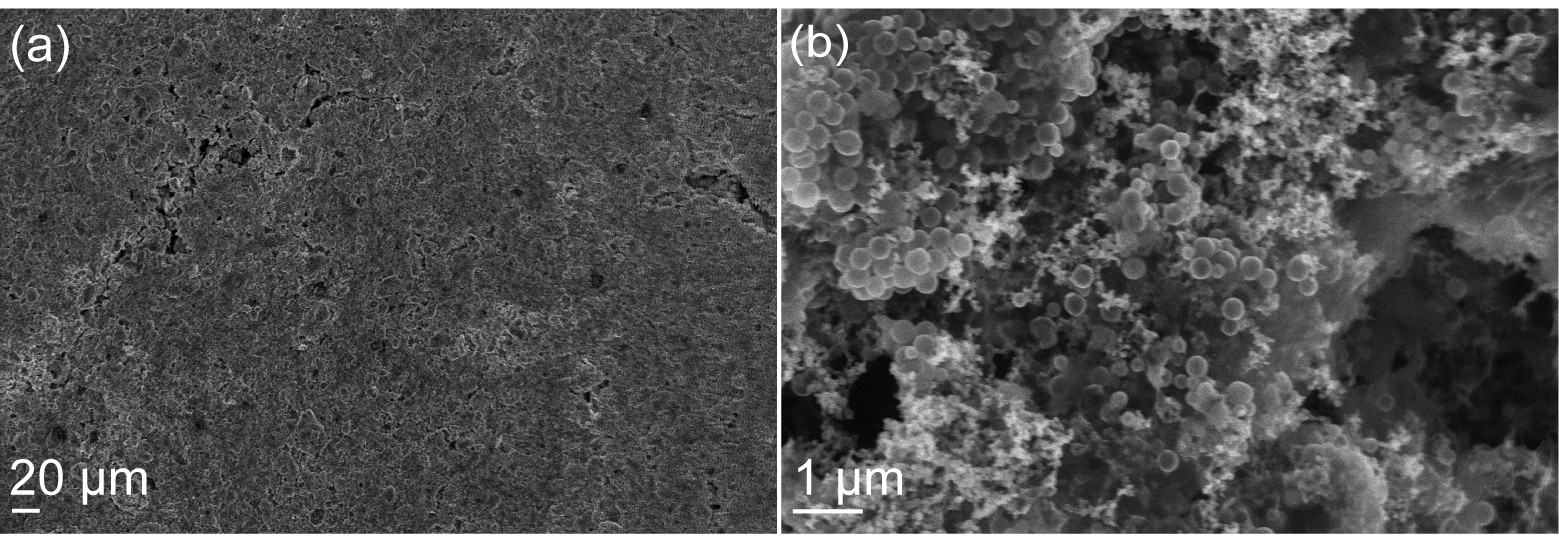


**Figure S16.** SEM images of the SMOP-S-HCS positive electrode containing 6 wt% SMOP at (a) low and (b) higher magnification.


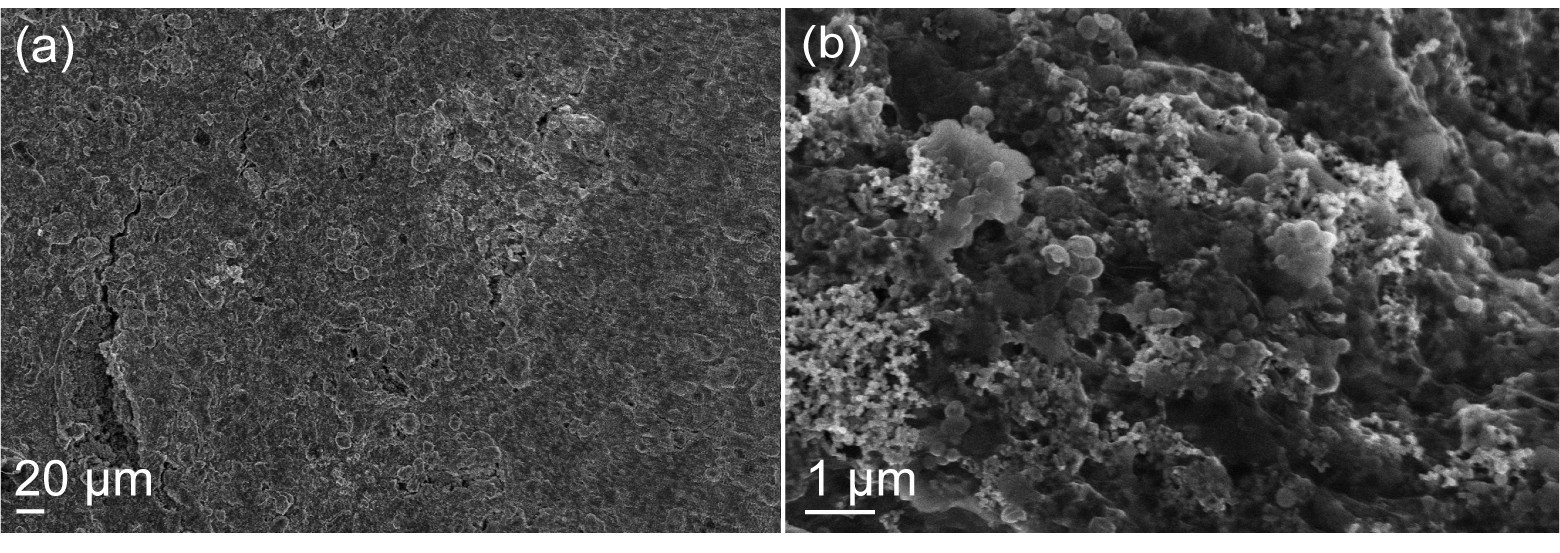


**Figure S17.** SEM images of the SMOP-S-HCS positive electrode containing 9 wt% SMOP at (a) low and (b) higher magnification.

**
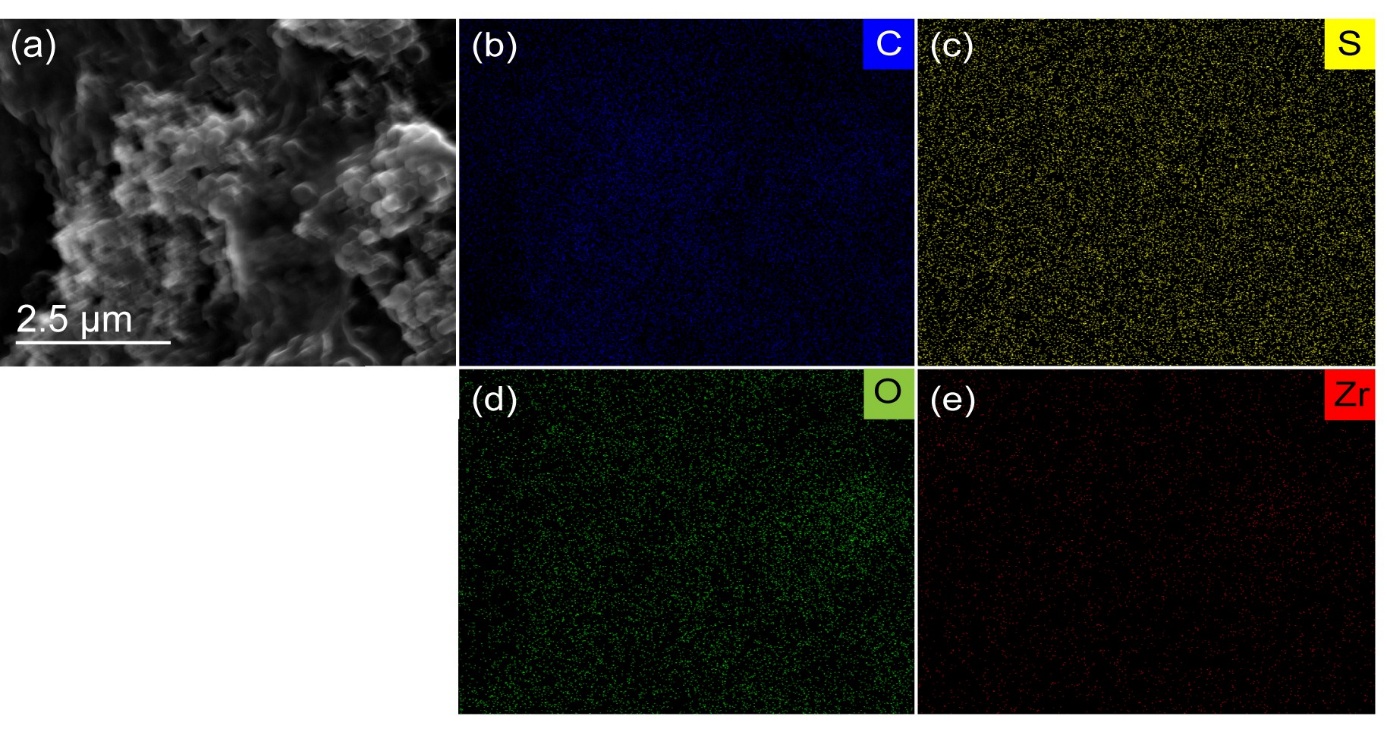
**

**Figure S18.** Post-cycling SEM-EDS characterization of the SMOP-S-HCS positive electrode after 100 cycles at 0.1 C. Backscattered electron image and corresponding elemental mapping results of C, O, S, and Zr.
